# Bottom-Up Assembly from a Helicate to Homochiral Micro- and Mesoporous Metal–Organic Frameworks**

**DOI:** 10.1002/anie.201004885

**Published:** 2010-12-29

**Authors:** Xiaobing Xi, Yu Fang, Taiwei Dong, Yong Cui

**Keywords:** bottom-up assembly, chirality, metal–organic frameworks, porosity, supramolecular chemistry

Stepwise assembly has emerged as a powerful technique to organize modular building blocks into target frameworks, whose topologies and functions may be dictated by the geometry and chemical functionality of the molecular constituents.^1^ This bottom-up approach not only offers an efficient approach to target hybrid materials with minimal effort, but also provides insight into the mechanism of the assembly process.^2^ Metal–organic frameworks (MOFs) provide an intriguing way to design hybrid materials from organic struts and metal ions, and have attracted great attention because of their fascinating structures and potential applications in diverse areas.^3^ With few exceptions, however, MOFs are always fabricated by a one-pot procedure.^4^

Helical structures are integral to myriad highly sophisticated bioarchitectures,^5^ which have motivated chemists to make artificial helical structures.^6^ In particularly, as a result of their intrinsic chirality, nanoscale shapes, and rich physicochemical properties, helicates constructed from flexible oligodentate strands and metal ions have been shown to be superb molecular systems in the bottom-up assembly of smart materials and devices.^7^, ^8^ Although helicates can, in principle, be designed to have predictable geometries and functional groups that can participate in coordination interactions, there is no report on the stepwise assembly of helicates or helices into a MOF.^3^, ^4^

We recently showed that *C*_2_-symmetrical 1,1′-biphenol derivatives are excellent platforms for creating helical species.^9^ Our strategy for making helicate-based ligands consists of using a tetraanionic hexadentate 1,1′-biphenol ligand bearing two pyridine-functionalized Schiff base units at the *ortho* positions. A pair of terminal NO donors may chelate metal ions to form linear helicates, and the two pendant biphenolic oxygen atoms may entrap more metal ions into the helical cavity, thereby leading to a cluster structure with free pyridyl groups. We report here the synthesis of a pyridyl-functionalized triple-stranded heptametallic helicate, and show that it can be used as a building block for the stepwise assembly of homochiral micro- and mesoporous MOFs through supramolecular interactions or coordination bonds

The enantiopure Schiff base ligand (MOM)_2_L−2 H was synthesized from 5,5′,6,6′-tetramethyl-2,2′-diol-1,1′-biphenyl in four steps in an overall yield of 39 % (Scheme 1). The reaction of (*R*)-(MOM)_2_L−2 H and CuSO_4_⋅5 H_2_O (1:2 molar ratio) in DMSO and 2-BuOH at 80 °C afforded [Cu_7_(OH)_2_L_3_]⋅2 DMSO⋅2 H_2_O (**1**). The product is soluble in DMSO and practically insoluble in water and other common organic solvents. Heating **1** and CuSO_4_⋅5 H_2_O (1:2 molar ratio) in DMSO afforded [{Cu_7_(OH)_2_L_3_}{Cu_6_(OH)_2_(SO_4_)_3_(S_3_O_10_)_2_}]⋅10 H_2_O (**2**) at 80 °C and [{Cu_7_(OH)_2_L_3_}_2_{Cu_6_(OH)_2_(SO_4_)_6_(S_2_O_7_)}{Cu_3_(SO_4_)(H_2_O)_6_}]⋅18 H_2_O (**3**) at 100 °C. Complexes **2** and **3** are stable in air and insoluble in water and organic solvents, and were formulated on the basis of elemental analysis as well as IR and thermogravimetric analysis (TGA). The phase purity of the bulk samples of **1**–**3** was established by comparison of their observed and simulated powder X-ray diffraction (PXRD) patterns.

**Scheme 1:**
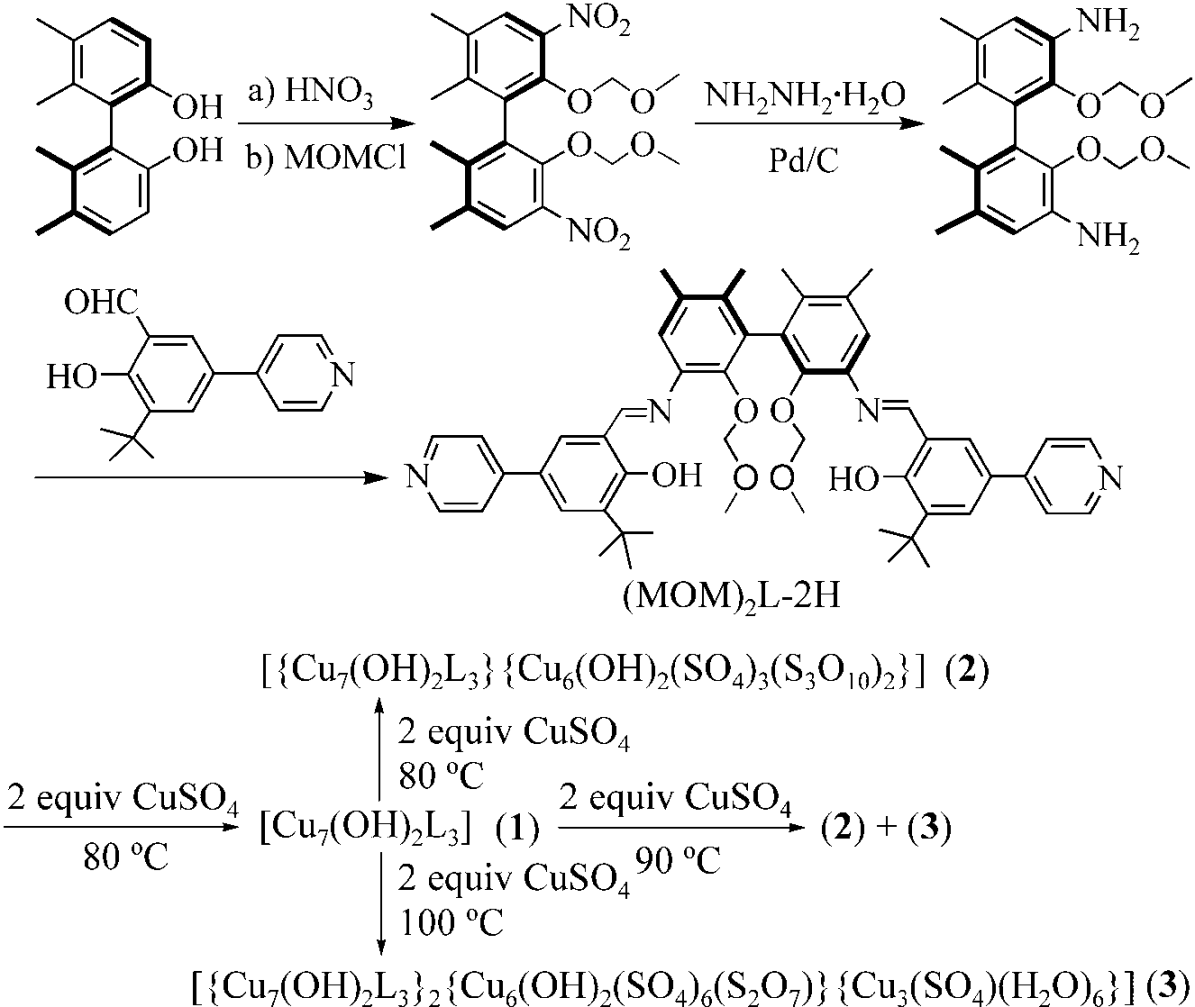
Synthesis of the ligand (MOM)_2_L−2 H and the MOFs. MOM=methoxymethyl.

A single-crystal X-ray diffraction study on **1** reveals a heptanuclear helical structure that crystallizes in the chiral trigonal space group *P*3_2_21 with one formula unit in the asymmetric unit (Figure 1).^10^ Seven metal ions are engaged in two distorted Cu_4_O_4_ cubanes by sharing one Cu ion. The six outer Cu ions are each square-pyramidally coordinated by one OH^−^ ion as well as one N atom and three O atoms from two L ligands, while the central Cu ion is octahedrally coordinated to three N and three O atoms from three L ligands. The MOM groups were completely removed from the starting ligands upon complexation with the metal ions, and each ligand L binds to two metal ions through two tridentate NO_2_ donors and to another two metal ions through two biphenolate oxygen atoms. Such an arrangement of the dicubane unit and the three L ligands leads to a *P*-configured triple-stranded helicate. With one crystallographic *C*_3_ axis running through a pair of μ_3_-O atoms and three crystallographic *C*_2_ axes that bisect three pairs of opposite L edges, the Cu_7_ helicate possesses *D*_3_ point group symmetry.

**Figure 1:**
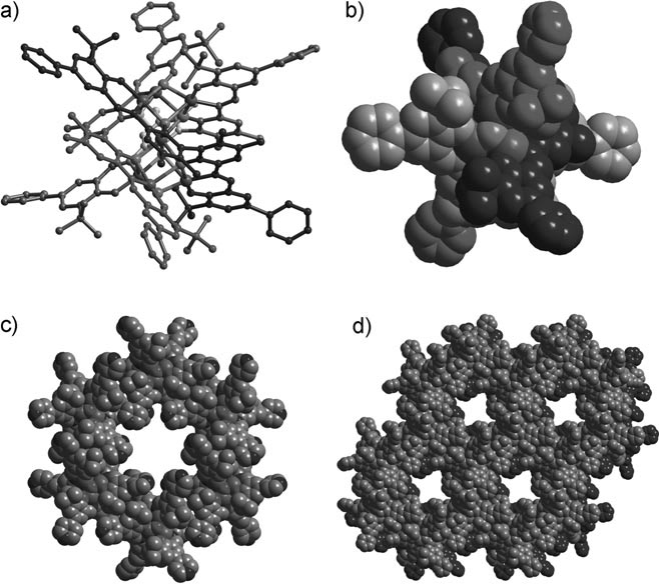
a) The helical structure of **1** and b) its space-filling mode. c) A macrocycle assembled from six helicates and d) the 3D porous structure of **1** viewed along the *b* axis.

Strong CH⋅⋅⋅π interactions between the methyl group and the conjugated pyridine ring of adjacent helicates (C–H⋅⋅⋅π=2.65–3.86 Å) direct the packing of helicates along the *c* axis, thereby making a nanosized tubule with an opening of 1.2×1.1 nm. The supramolecular structure is reinforced by hydrophobic interactions between *tert*-butyl groups of adjacent helicates and face-to-face intermolecular π–π interactions (plane-to-plane separation=3.82 Å; see Figure S3 in the Supporting Information). Highly directional noncovalent interactions in **1** have thus clearly steered the packing of the helicates to make a 3D nanotubular architecture (Figure 1d). The peripheral free pyridyl groups of **1** may potentially coordinate additional metal ions to construct extended structures.

Complex **2** crystallizes in the chiral hexagonal space group *P*6_3_22. The Cu_7_ helicate binds to six newly generated [Cu_6_(μ_3_-OH)_2_(μ_2_-SO_4_)_3_(μ_3_-S_3_O_10_)_2_] (Cu_6_-α) clusters through pyridyl groups. In this Cu_6_-α cluster, the metal centers form a *D*_3_-symmetrical trigonal prism with the top and bottom faces bridged by two μ_3_-S_3_O_10_ anions and the other three faces by three μ_2_-SO_4_ anions; the six-coordinate, octahedral geometry at each metal center is completed by a μ_3_-OH unit or a pyridyl group. Therefore, each Cu_6_ cluster binds to six pyridyl groups of six Cu_7_ helicates, and each Cu_7_ helicate connects six Cu_6_-α clusters to form a (6,6)-connected network.

Six Cu_7_ clusters and five Cu_6_-α clusters that are related by *C*_3_ symmetry merge to generate a *D*_3_-symmetric 4^6^3^6^-α cage. The cage has an open spherical cavity with an internal diameter of 2.36 nm (considering van der Waals radii) which is occupied by a disordered guest molecule (Figure 2), while the quadrilateral aperture on each face has diagonal distances of approximately 1.6×1.6 nm. The cage shares its quadrilateral and triangular faces with 12 neighboring cages, while the sharing of the square faces gives rise to multidirectional zig-zag channels in the framework of **2**.

**Figure 2:**
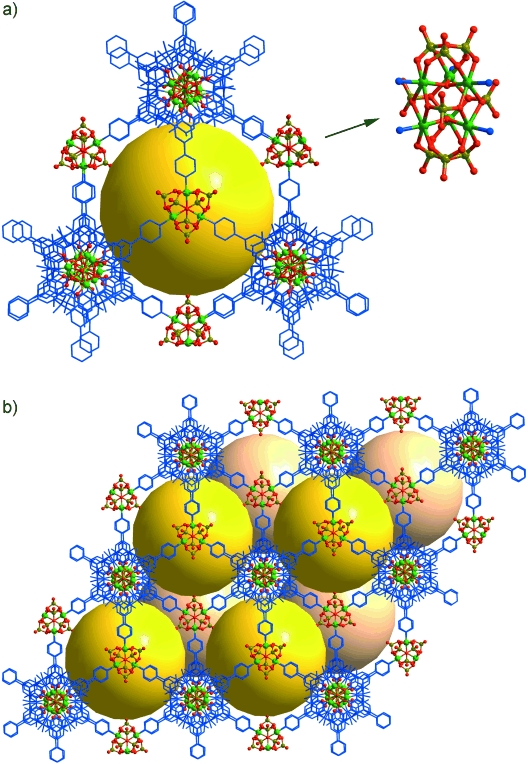
a) A mesoporous cage in **2** constructed of six [Cu_7_(OH)_2_L_3_] helicates and five [Cu_6_(OH)_2_(SO_4_)_3_(S_3_O_10_)_2_] clusters. b) The 3D porous structure of **2** viewed along the *c* axis.

Complex **3** also crystallizes in the chiral hexagonal space group *P*6_3_22. However, the six pyridine rings of each helicate are alternatively linked by two types of *D*_3_-symmetrical metal clusters, namely, a SO_4_^2−^-bridged trimetal cluster [Cu_3_(μ_3_-SO_4_)(H_2_O)_6_] and a hexanuclear cluster [(Cu_3_(μ_3_-OH)(μ-SO_4_)_3_)_2_(μ_6_-S_2_O_7_)] (Cu_6_-β) with two triangular {Cu_3_(μ_3_-OH)} units bonded by three μ-SO_4_^2−^ ions and linked through one μ_6_-S_2_O_7_^2−^ ion (Figure 3). In the two cases the five-coordinate trigonal-bipyramidal geometry at each metal center is completed by two pyridine ligands and two water molecules, and by one pyridine ring, respectively. Both the hexa- and tricopper clusters are six-connected nodes linked by six pyridyl groups of helicate **1**, and each helicate **1** bridges three Cu_3_ clusters and three Cu_6_-β clusters in a hexadentate fashion, thereby generating a (6,6)-connected framework.

**Figure 3:**
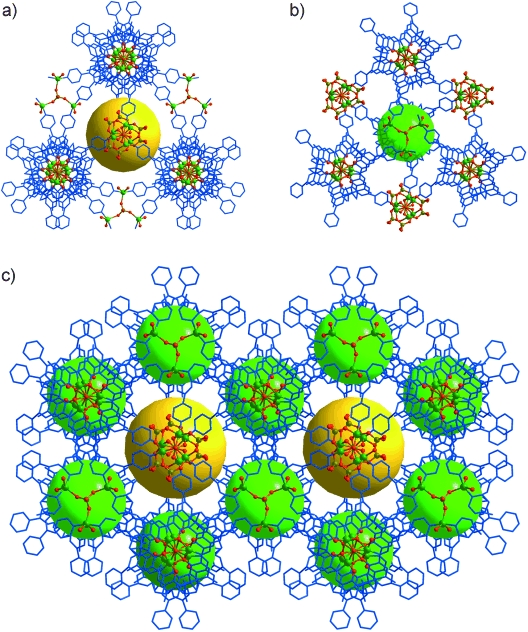
a) A mesoporous cage in **3** constructed of six [Cu_7_(OH)_2_L_3_] helicates, three [Cu_3_(SO_4_)(H_2_O)_6_] clusters, and two [Cu_6_(OH)_2_(SO_4_)_6_(S_2_O_7_)] clusters. b) A mesoporous cage in **3** constructed of four [Cu_7_(OH)_2_L_3_] helicates, one [Cu_3_(SO_4_)(H_2_O)_6_] cluster, and three [Cu_6_(OH)_2_(SO_4_)_6_(S_2_O_7_)] clusters. c) The 3D porous structure of **3** viewed along the *c* axis.

The framework of **3** consists of two types of *D*_3_-symmetrical cages, namely a larger 4^6^3^6^-β cage, similar to that in **2**, encapsulated by six Cu_7_ clusters, three [Cu_3_(SO_4_)(H_2_O)_6_] and two Cu_6_-β clusters, as well as a smaller 4^6^ cage enclosed by four Cu_7_ helicates, one [Cu_3_(SO_4_)(H_2_O)_6_], and three Cu_6_-β clusters. Each type of cage has an irregular cavity that has a maximum inner width of approximately 2.3 and 1.8 nm, respectively, and is occupied by guest molecules. The quadrilateral aperture on each face has a diagonal distance of approximately 1.6×1.4 nm. The 4^6^3^6^-β cage shares its square and triangular faces with six 4^6^ cages and six 4^6^3^6^-β cages, respectively, while the 4^6^ cage shares its quadrilateral faces with three 4^6^ cages and three 4^6^3^6^-β cages. Sharing of the quadrilateral windows with neighboring cages leads to multidirectional zig-zag channels in the framework of **3**.

Helicate **1** is stable in DMSO, as shown by ESI-MS, which gave a prominent signal for [Cu_7_(OH)_2_**L**_3_+7 H]^7+^ at *m*/*z*=386.9. The UV/Vis spectra of **1** in DMSO at room temperature, 80 °C, and 100 °C showed identical absorption bands at 320, 432, 459, and 605 nm. The CD spectra of solutions of **1** in DMSO are also similar at room and elevated temperatures (Figures S19 and S21). Taken together, these results indicate that the helical structure and the optical activity of **1** are maintained without any apparent change while assembling into frameworks in solution. To our knowledge, this is the first example of a truly stepwise construction of MOFs by using a helicate. The self-assembly and amplification of intrinsic information encoded in the Cu_7_ helicate is expressed by the formation of the Cu_6_-α and -β and Cu_3_ clusters, and finally the three types of assembled 4^6^3^6^-α and -β and 4^6^ cages in **2** and **3**, which have the same handedness of chirality and *D*_3_ symmetry as the helicate precursor. Thus, the coordination-driven stepwise assembly of helicate **1** enabled its geometry, symmetry, and enantiopurity to be amplified highly efficiently in the infinite frameworks.

Temperatures of 80 and 100 °C promote formation of different clusters, cages, and frameworks, the Cu_7_ helicate precursors of which all have *D*_3_ symmetry. In particularly, the Cu_6_-β and Cu_3_ clusters in **3** may be viewed as originating from partial and complete decomposition of Cu_6_-α units in **2** at the elevated temperature. A mixture of **2** and **3** was obtained at the intermediate temperature of 90 °C. New phases that have yet to be identified were obtained at higher and lower temperatures. The role of temperature in controlling the assembly process may be rationalized, as higher temperatures would naturally be expected to afford more thermodynamically stable and denser crystal forms.^11^

The solid-state CD spectra of **1**–**3** made from *R* and *S* enantiomers of L are mirror images of each other, thus indicating their enantiomeric nature. Calculations using PLATON indicate that 39.8, 59.0, and 45.2 % of the total volume of **1**–**3**, respectively, are occupied by solvent molecules.^12^ TGA revealed that the solvent molecules could be removed from them in the 50–130 °C range. Powder XRD experiments indicate that the three frameworks retain their structural integrity and crystallinity upon removal of the guest. Their permanent porosities were confirmed by their N_2_ adsorption isotherms at 77 K and by liquid-phase adsorptions. Helicate **1** exhibits a type I sorption behavior, with a BET surface area of 365 m^2^ g^−1^, whereas **2** and **3** exhibit type IV sorption behaviors, with BET surface areas of 375.1 and 421.4 m^2^ g^−1^, respectively (Figures S22–24). The observed surface areas for **2** and **3** are clearly smaller than the theoretical values of 1570.0 and 1084.0 m^2^ g^−1^ for **2** and **3**, respectively,^13^ which is indicative of the distortion of the frameworks upon removal of the guest molecules.

Interestingly, **2** and **3** could readily adsorb 4.32 and 4.97 Rhodamin 6G molecules (ca. 1.4 nm×1.6 nm in size) and 1.12 and 1.25 Brilliant Blue R-250 molecules (1.8 nm×2.2 nm in size) per formula unit, respectively. These guest-included solids exhibited the same PXRD patterns as the pristine **2** and **3**. These results indicate that the structural integrity and open channels of these mesoporous MOFs are maintained in solution. The synthesis of MOFs with mesoporosity remains a great challenge because of their tendency to reduce or eliminate porosity through interpenetration or other void-filling means^14^ and crystals of mesoporous MOFs tend to disintegrate upon removal of the guest.^15^ As a result, only a few mesoporous MOFs have been reported.^15^, ^16^ Chiral mesoporous MOFs with permanent porosity and large open channels are even more scarce.^16^, ^17^ Moreover, all of them exhibit straight tubular channels, whereas **2** and **3** are characteristic of zeolitic topologies with large cages and small apertures, combine the common features of traditional zeolites and MOFs, and may be expected to be advantageous for enantioselective recognition and catalysis.^17^, ^18^

In conclusion, we have described the step-by-step assembly of three homochiral micro- and mesoporous MOFs from a predesigned triple-stranded helicate bearing hierarchical functional groups. Compounds **2** and **3** represent the first two mesoporous zeolite-like MOFs to be reported.^17^ The initial results on gas and liquid adsorption provide insight into the potential of these materials in inclusion chemistry. Work is in progress to explore the potential of constructed MOFs as hosts for molecules with applications in enantioselective processes. Given the high structural diversity of helicates, this work opens up new perspectives for the hierarchical assembly of fascinating chiral networks.

## Experimental Section

**1**: A mixture of CuSO_4_⋅5H_2_O (25 mg, 0.1 mmol) and (MOM)_2_L−2 H (41.7 mg, 0.05 mmol) was placed in a small vial containing DMSO (1 mL), H_2_O (0.1 mL), and *s*BuOH (1 mL). The vial was sealed and heated at 80 °C for one day. Turquoise rodlike crystals of **1** were collected, washed with diethyl ether, and dried in air. Yield: 33.9 mg (80 % based on Cu). Elemental analysis (%): calcd for C_148_H_156_Cu_7_N_12_O_18_S_2_: C 61.30, H 5.42, Cu 15.34, N 5.80, S 2.21; found: C 60.20, H 5.39, Cu 15.24, N 5.76, S 2.20. ESI-MS: *m*/*z* 2707.5 (calcd *m*/*z* 2708.6 for [*M*+H]^+^).

**2**: A mixture of CuSO_4_⋅5H_2_O (25 mg, 0.1 mmol) and **1** (135 mg, 0.05 mmol) was placed in a small vial containing DMSO (1 mL), H_2_O (0.1 mL), and *s*BuOH (1 mL). The vial was sealed, heated at 80 °C for one day, and the turquoise block-like crystals of **2** were collected, washed with diethyl ether, and dried in air. Yield: 57.0 mg, 75 % based on Cu. Elemental analysis (%): calcd for C_144_H_162_Cu_13_N_12_O_58_S_9_: C 42.15, H 3.98, Cu 20.13, N 4.10, S 7.03; found: C 41.97, H 3.91, Cu 20.02, N 4.05, S 6.97.

**3**: The procedure was as for **2**, and the vial was sealed, heated at 100 °C for one day. The turquoise block-like crystals of **3** were collected, washed with diethyl ether, and dried in air. Yield: 56.4 mg, 60 % based on Cu. Elemental analysis (%): calcd for C_288_H_330_Cu_23_N_24_O_89_S_9_: C 47.37, H 4.56, Cu 20.02; N 4.60, S 3.95; found: C 47.24, H 4.49, Cu 19.97, N 4.56, S 4.00.

The dye-inclusion experiment: Fresh crystal samples of **2** (3 mg) and **3** (3 mg) were soaked in a solution of Rhodamine 6G (60 mm) in methanol for 12 h. The red crystals were washed with water thoroughly until the filtrate became colorless. The solids were digested with Na_2_EDTA (0.05 m, 2 mL) and NaOH (6 m, 0.1 mL), and then the resultant clear solution with a light red color was diluted to 100 mL. The same procedures were also used for the Brilliant Blue R-250 uptake studies. The concentrations of the dyes were determined by comparing the UV/Vis absorptions with the standard curves.
