# Supplementary material for: Bottom-Up Assembly from a Helicate to Homochiral Micro- and Mesoporous Metal–Organic Frameworks
Source: Angew Chem Int Ed Engl. 2010 Dec 29;50(5):1154–8. doi: 10.1002/anie.201004885 (PMC3047008; doi:10.1002/anie.201004885)

Angewandte Chemie
Eine Zeitschrift der Gesellschaft Deutscher Chemiker

Supporting Information
© Wiley-VCH 2011
69451 Weinheim, Germany

Bottom-Up Assembly from a Helicate to Homochiral Micro- and Mesoporous Metal–Organic Frameworks**

*Xiaobing Xi, Yu Fang, Taiwei Dong, and Yong Cui**

anie_201004885_sm_miscellaneous_information.pdf

Table of Content

1. Materials and general procedures
2. Synthesis of the ligand **L-**(2MOM-2H) and IR data for compound **1**-**3**
3. **Table S1**. Summary of dye uptake by MOFs.
4. **Table S2.** Crystal data and structure refinement
5. **Tables S3-S5.** Selected Bond lengths [Å] and angles [°] for **1-3**
6. **Figures S1** – **S4**. Additional X-ray crystallographic structures of compound **1**
7. **Figures S5-S9**. Additional X-ray crystallographic structures of compound **2**
8. **Figures S10-S14**. Additional X-ray crystallographic structures of compound **3**
9. **Figures S15-S17**. PXRD patterns of compounds **1-3**
10. **Figure S18**. TGA curves of compounds **1, 2** and **3**
11. **Figure S19**. CD spectra of (*R*)/(*S*)-**L-**(2MOM-2H)**, -1, -2** and **-3**
12. **Figure S20**. UV/Vis absorption spectra of **1-3** and **L-**(2MOM-2H) in the solid state
13. **Figure S21**. UV/Vis absorption spectra of **1** in DMSO at r.t., 80 and 100 ^{o}C
14. **Figures S22-24.** The N_2 adsorption isotherms for **1-3**
15. **Figures S25** and **26.** Mass spectra of the ligand **L**-(2MOM-2H), related organic compounds and the helicate **1**
16. **Figures S27** and **28.** ^{1}H and ^{13}C NMR spectra of the ligand **L**-(2MOM-2H) and related organic compounds.
17. **Figures S29.** UV-Vis Absorption spectra of digested frameworks **2** and **3** loaded with Rhodamine 6G and Brilliant Blue R-250.
18. **Figure S30.** UV-Vis Absorption spectra of Rhodamine 6G and Brilliant Blue R-250. (standard solution)
19. **Figure S31.** Calibration curve for UV-Vis absorption of Rhodamine 6G and Brilliant Blue R-250.
20. **Figures S32.** Powder X-ray diffraction patterns of **2** and **3** with and without Rhodamine 6G and Brilliant Blue R-250.

1**. Materials and General Procedures.**

All of the chemicals are commercial available, and used without further purification. Elemental analyses were performed with an EA1110 CHNS-0 CE elemental analyzer. Inductively coupled plasma atomic emission spectroscopy (ICP-AES) was performed with a BAIRD ICP 2000 Emission Spectrometer. The IR (KBr pellet) spectrum was recorded (400-4000 cm^{-1} region) on a Nicolet Magna 750 FT-IR spectrometer. The N_2 adsorption isotherms was measured at 77 K by using a Micromeritics ASAP 2020 system. The CD spectra were recorded on a J-800 spectropolarimeter (Jasco, Japan). Thermogravimetric analyses (TGA) were carried out in an air atmosphere with a heating rate of 10 ^{o}C/min on a STA449C integration thermal analyzer. Powder X-ray diffraction (PXRD) data were collected on a DMAX2500 diffractometer using Cu Kα radiation. The calculated PXRD patterns were produced using the SHELXTL-XPOW program and single crystal reflection data. All UV/Vis absorption spectrum were recorded on a Lambda 20 UV/Vis Spectrometer(Perkin Elmer, Inc., USA). ^{1}H and ^{13}C NMR experiments were carried out on a MERCURYplus 400 spectrometer operating at resonance frequencies of 100.63 MHz. Electrospray ionization mass spectra (ES-MS) were recorded on a Finnigan LCQ mass spectrometerusing dichloromethane-methanol as mobile phase.

X-ray Crystallography. Single-crystal XRD data for compounds **1**-**3** were all collected on a Bruker SMART Apex II CCD-based X-ray diffractometer with Cu-Kα radiation (λ = 1.54178 Å) at 123 K. The empirical absorption correction was applied by using the SADABS program (G. M. Sheldrick, SADABS, program for empirical absorption correction of area detector data; University of Göttingen, Göttingen, Germany, 1996). The structure was solved using direct method, and refined by full-matrix least-squares on F2 (G. M. Sheldrick, SHELXTL97, program for crystal structure refinement, University of Göttingen, Germany, 1997). In all compounds, the guest molecules and H-atoms were refined isotropically, while all other atoms were refined anisotropically. The single-crystal diffraction showed that the products have the formula $[Cu_7(OH)_2\mathbf{L}_3]$ 2DMSO $6H_2O$ (**1**), $[Cu_7(OH)_2\mathbf{L}_3][Cu_6(OH)_2(SO_4)_3$ $.(S_3O_{10})_2]$ $34H_2O$ (**2**) and $[Cu_7(OH)_2\mathbf{L}_3]_2[Cu_6(OH)_2(SO_4)_6(S_2O_7)][Cu_3(SO_4)(H_2O)_6$ $30H_2O$ (**3**). Crystal data and details of the data collection are given in Table S2, while the selected bond distances and angles are presented in Tables S3-S5.

2. Synthesis of the ligand L-(2MOM-2H)

2.1 Synthesis of (*R*)-3,3’-Dinitryl-5,5’,6,6’-Tetramethyl-1,1’-biphenyl-2,2’-diol**:**

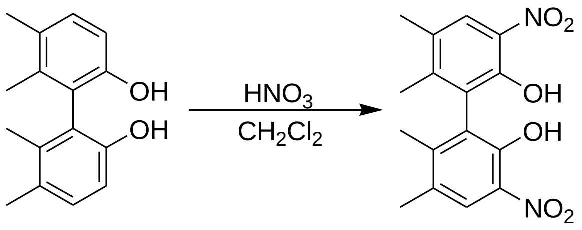

5,5’,6,6’-Tetramethyl-1,1’-biphenyl-2,2’-diol (1.5 g, 6.18 mmol) was dissolved in dichloromethane (140 mL) in a 250 ml round bottomed flask. 1mL of concentrated nitric acid (24.6 mmol, 65%) was added dropwise to the solution and the reaction mixture was stirred 5h, while keeping the temperature below 30 ℃. Then the solvent was evaporated in vacuo, the residue was purified by flash chromatography (silica gel, hexane: ethyl acetate = 40:1) to yield the product (1.6 g, 78% yield) as a yellow solid after evaporation of the solvent; 1H NMR ($CDCl_3$, TMS, 400MHz): 10.764(s, 2H, OH), 7.994(s, 2H, ArH), 2.348(s, 6H, Me), 2.001(s, 6H, Me); 13C NMR (CDCl3, TMS, 400MHz): 151.245, 148.142, 131.717, 129.622, 126.115, 124.870, 20.092, 17.677. ESI-MS: m/z 331.1 (Calcd *m/z* 332.1 for $[M+H]^+$) (see Figure **S25**).

2.2 Synthesis of (*R***)**-3,3’-Dinitryl-5,5’,6,6’-tetramethyl-2,2’-methoxymethyl-1,1’-biphenyl:

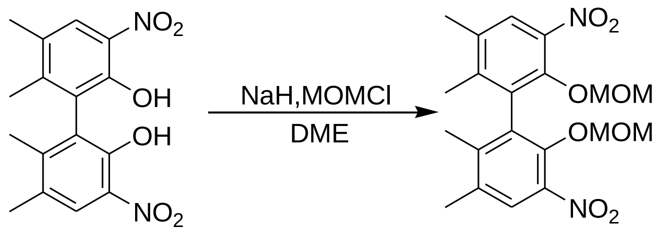

Under a nitrogen atmosphere, 3,3’-Dinitryl-5,5’,6,6’-Tetramethyl-1,1’-biphenyl- 2,2’-diol (1.45 g, 4.37 mmol) was added to asuspension of NaH (1.75 g, 72.9 mmol) dropwise in anhydrous DME (100 mL) at room temperature with stirring. The resulting solution was stirred for 30 min, and heated to 55 ℃. After the solution turned to dark red,methoxymethyl chloride (13.6 mL, 48 mmol) was slowly added. The mixture was allowed to stay at this temperature and stirred for 2 h. Then the reactionwas quenched by water. after evaporation of the DME, the aqueous layer was extracted with ethyl acetate (2 × 100 mL). The combined organic layers were washed with brine and dried over $MgSO_4$. After removal of the solvent, the product as a white solid was obtained (1.5 g, 81.7% yield), which was pure enough for the next step. ^{1}H NMR ($CDCl_3$, TMS, 400MHz): 7.762(s, 2H, ArH), 4.836(s, 4H, CH_2), 2.893(s, 6H, Me), 2.363(s, 6H, Me), 1.990(s, 6H, Me); 13C NMR ($CDCl_3$, TMS, 400MHz): 147.591, 143.952, 141.890, 133.834, 133.689, 125.862, 101.158, 56.731, 20.284, 17.915 ESI-MS: m/z 420.1 (Calcd *m/z* 438 for $[M+ NH_4^+]$) (see Figure **S25**).

2.3 Synthesis of (*R*)-3,3'-diamino-5,5',6,6'-tetramethyl-2,2'- methoxymethyl -1,1'-biphenyl:

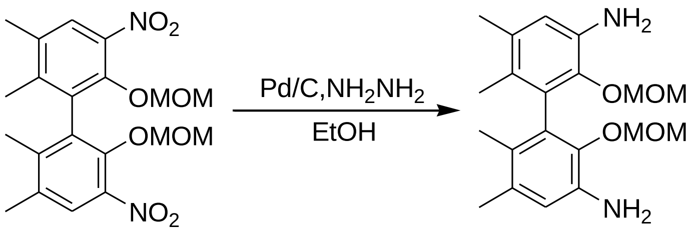

A 250 mL round bottom flask was placed under a nitrogen atmosphere and charged with the 3,3'-Dinitryl-5,5',6,6'-tetramethyl-2,2'- methoxymethyl -1,1'-biphenyl (2.12 g, 5.03 mmol), Palladium on charcoal (10%, 462 mg), hydrazine hydrate (128.3 mL, 1.69 mol) and EtOH(250 mL), the reaction mixture was refluxed and stirred 12h. The cooled mixture was filtered off and the filtrate was concentrated to give product as colorless liquid (1.37 g, 75.7%); ^{1}H NMR ($CDCl_3$, TMS, 400MHz): 6.605(s, 2H, ArH), 4.587(s, 4H, CH_2), 3.142(s, 6H, Me), 2.185(s, 6H, Me), 1.819(s, 6H, Me); ^{13}C NMR ($CDCl_3$, TMS, 400MHz): 140.785, 137.618, 132.988, 132.342, 125.604, 117.224, 98.465, 56.832, 20.356, 16.141 ESI-MS: m/z 361.2 (Calcd *m/z* 360 for $[M+H]^+$) (see Figure **S25**).

2.2 Synthesis of (*R*)-*N,N*'-Bis(3-*tert*-butyl-5-(4 -pyridyl) salicylidene)-3,3' -diamino -5,5',6,6'- tetramethyl-2,2'-methoxymethyl -1,1'-biphenyl (**L-(2MOM-2H**):

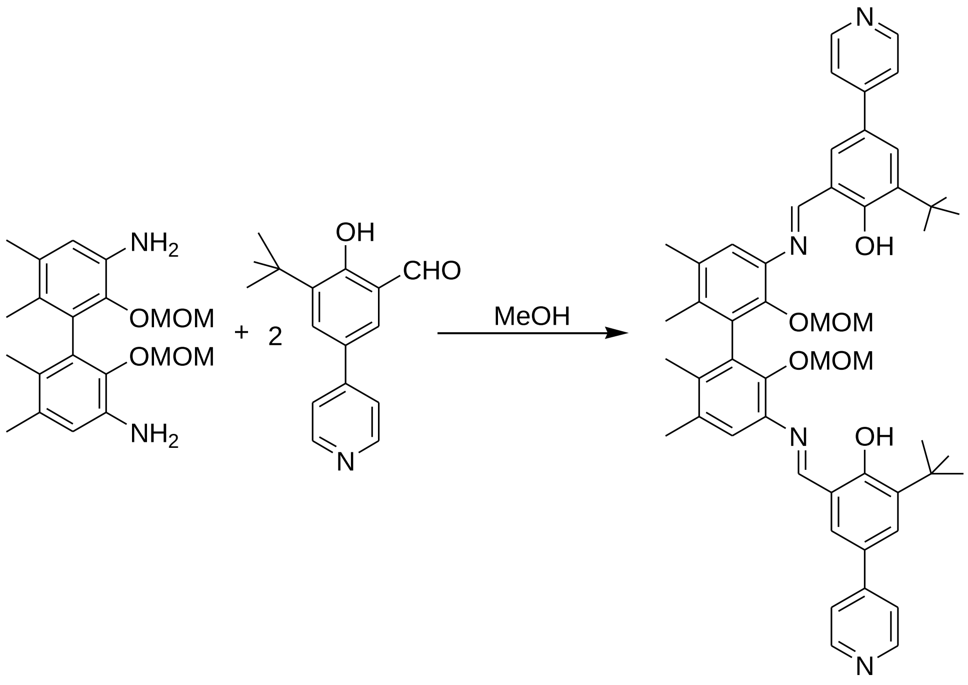

A mixture of 3,3'-diamino-5,5',6,6'-tetramethyl-2,2'- methoxymethyl -1,1'-biphenyl (2.6 g, 10 mmol) and 3-tert-butyl-5-(4-pyridyl) salicylaldehyde (5.13g, 20.1 mmol) in MeOH (80 mL) was refluxed and stirred for 24 h, and the reaction mixture was then filtered to collect the product as an orange solid (6.6 g, 80%);^{1}HNMR ($CDCl_3$) δ:8.82 (s, 2H, HC=N) 8.36 (d, 4H, pyridylH) , 7.66 (d, 2H, ArH) ,7.58 (d, 2H, ArH) , 7.5 (d, 4H, pyridylH) , 7.11 (s, 2H, ArH) , 4.85 (dd, 4H, CH_2) , 2.9 (s, 6H, Me), 2.36 (s, 6H, Me), 2.01 (s, 6H, Me) ,1.49 (s, 18H, CMe_3).

^{13}CNMR ($CDCl_3$) δ:162.86, 161.88, 150.42, 148.20, 147.47, 139.03, 138.76, 136.31, 133.58, 133.32, 129.06, 128.93, 127.97, 121.17, 120.22, 119.72, 99.71, 56.50, 35.38, 29.47, 20.56, 17.24. MALDI-TOF: m/z 834.4 (Calcd *m/z* 835.5 for $[M+H]^+$) (see Figure **S26**).

2.3. IR data for **1-3**

IR (KBr) for **1**: IR (KBr): 3423 (w), 2994 (m), 2918 (w), 2858 (w), 2326 (s), 1592 (s), 1503 (m), 1460 (m), 1384 (w), 1286 (m), 1259 (w), 1228 (m), 1025 (w), 897 (w), 829 (w), 790 (w), 705 (w), 616 (w), 531 cm^{-1} (w).

IR (KBr) for **2**: 3418 (w), 2950 (m), 2862 (w), 2364 (w), 1627 (s), 1599 (s), 1524 (m), 1443 (m), 1389 (w), 1362 (m), 1290 (w), 1219 (m), 1134 (w), 1020 (w), 973 (w), 864 (w), 778 (w), 729 (w), 524 cm^{-1} (w).

IR (KB) for **3**: 3510 (w), 2994 (m), 2910 (w), 2356 (w), 2334 (s), 1593 (s), 1503 (m), 1433 (m), 1381 (w), 1360 (m), 1257 (w), 1224 (m), 1109 (w), 1027 (w), 895 (w), 855 (w), 644 (w), 616 (w), 500 cm^{-1} (w)

3. ***Table S1***. Summary of dye uptake by MOFs **2** and **3** in MeOH.

	**2** + Rdm6G	**3** + Rdm6G	**2** + BBR250	**3** + BBR250
The number of dye uptake (per formula**)**	4.32	4.97	1.12	1.25
wt% dye/framework	45.6%	32.7%	21.4%	14.2%

4. *Table S2*. Crystal data and structure refinement for compounds **1-3**

Identification code	**1**	**2**	**3**
Empirical formula	$C_{148}H_{164}Cu_7N_{12}O_{22}S_2$	$C_{144}H_{210}Cu_{13}N_{12}O_{82}S_9$	$C_{144}H_{189}Cu_{11.5}N_{12}O_{50.5}S_{4.5}$
crystal system	trigonal	hexagonal	hexagonal
Formula weight	2965.66	4535.88	7517.91
T, K	123	123	123
Wavelength (Å)	1.54178	1.54178	1.54178
space group	$P3_221$	$P6_322$	$P6_322$
unit cell dimensions	a = 26.1935(2) Å<br>b = 26.1935(2) Å<br>c = 46.0311(6) Å<br>α = 90 º<br>β = 90 º<br>γ = 120 º	a = 26.2486(5) Å<br>b = 26.2486(5) Å<br>c = 25.2139(6) Å<br>α = 90 º<br>β = 90 º<br>γ = 120 º	a = 24.0343(2) Å<br>b = 24.0343(2) Å<br>c = 43.8206(7) Å<br>α = 90 º<br>β = 90 º<br>γ = 120 º
Volume ($Å^3$), Z	27350.7(5),	15044.7(5), 2	21921.6(4), 2
Density (calculated) (mg/m^3)	1.083	1.001	1.139
Absorption coefficient (mm^{-1})	1.545	2.073	2.112
F(000)	9282	4670	7738
θ range for data collection (°)	3.88 to 54.99	5.44 to 68.08	3.68 to 52.49
Limiting indices	-27 ≤ h ≤ 27, -27 ≤ k ≤ 22, -47≤ I ≤ 45	-31≤ h≤ 30, -24≤ k≤ 30, -27≤ l≤ 29	-19≤ h≤ 24, -24≤ k≤24, -42≤ I≤ 44
Reflns collected	82969	61273	43277
Independent reflections	21830 [R(int) = 0.0770]	8792 [R(int) = 0.0682]	7945 [R(int) = 0.0422]
Completeness to theta	54.99/95.9 %	68.08/95.0 %	52.49/94.1 %
Refinement method	Full-matrix least-squares on F^2	Full-matrix least-squares on F^2	Full-matrix least-squares on F^2
Data / restraints / parameters	21830 / 21 / 1390	8792 / 7 / 304	7945 / 18 / 500
Final *R* indices [I>2σ (I)]	R1 = 0.0872, wR2 = 0.2279	R1 = 0.0881, wR2 = 0.2184	R1 = 0.0883, wR2 = 0.2298
R indices (all data, F^2 refinement)	R1 = 0.01196, wR2 = 0.2558	R1 = 0.1081, wR2 = 0.2522	R1 = 0.1008, wR2 = 0.2445
GOF on F^2	1.105	1.146	1.147
Largest diff. peak and hole, e/$Å^3$	0.738 and -0.618	0.982 and -0.884	1.321 and -1.342
flack parameter	0.03(4)	0.01(5)	-0.01(6)

5.1. *Table S3.* Selected Bond lengths [Å] and angles [°] for **1.**

Cu(1)-O(9)	1.869(6)
Cu(1)-N(10)	1.910(8)
Cu(1)-O(13)	1.983(6)
Cu(1)-O(10)	1.983(6)
Cu(1)-O(7)	2.392(6)
Cu(2)-O(4)	1.883(6)
Cu(2)-N(3)	1.887(9)
Cu(2)-O(3)	1.955(6)
Cu(2)-O(14)	2.009(6)
Cu(2)-O(6)	2.346(6)
Cu(3)-O(7)	2.040(7)
Cu(3)-O(11)	2.081(7)
Cu(3)-O(2)	2.108(6)
Cu(3)-O(6)	2.113(6)
Cu(3)-O(10)	2.113(6)
Cu(3)-O(3)	2.136(7)
Cu(4)-O(8)	1.880(6)
Cu(4)-N(7)	1.930(9)
Cu(4)-O(13)	1.968(7)
Cu(4)-O(7)	2.002(6)
Cu(4)-O(2)	2.398(7)
Cu(5)-O(1)	1.890(7)
Cu(5)-N(2)	1.899(10)
Cu(5)-O(13)	1.982(7)
Cu(5)-O(2)	1.997(7)
Cu(5)-O(10)	2.429(6)
Cu(6)-O(5)	1.861(8)
Cu(6)-N(6)	1.891(9)
Cu(6)-O(6)	1.972(7)
Cu(6)-O(14)	2.010(7)
Cu(6)-O(11)	2.385(6)
Cu(7)-O(12)	1.877(7)
Cu(7)-N(11)	1.902(8)
Cu(7)-O(11)	1.988(6)
Cu(7)-O(14)	2.016(6)
Cu(7)-O(3)	2.363(7)
O(9)-Cu(1)-N(10)	95.3(3)
O(9)-Cu(1)-O(13)	97.0(3)
N(10)-Cu(1)-O(13)	157.7(3)
O(9)-Cu(1)-O(10)	172.1(3)
N(10)-Cu(1)-O(10)	85.0(3)
O(13)-Cu(1)-O(10)	85.4(3)
O(9)-Cu(1)-O(7)	93.6(3)
N(10)-Cu(1)-O(7)	121.2(3)
O(13)-Cu(1)-O(7)	76.5(2)
O(10)-Cu(1)-O(7)	79.6(3)
O(4)-Cu(2)-N(3)	95.8(3)
O(4)-Cu(2)-O(3)	175.1(3)

N(3)-Cu(2)-O(3)	85.2(3)
O(4)-Cu(2)-O(14)	96.3(3)
N(3)-Cu(2)-O(14)	152.7(3)
O(3)-Cu(2)-O(14)	84.9(3)
O(4)-Cu(2)-O(6)	94.9(3)
N(3)-Cu(2)-O(6)	126.1(3)
O(3)-Cu(2)-O(6)	80.7(3)
O(14)-Cu(2)-O(6)	77.0(2)
O(7)-Cu(3)-O(11)	171.3(3)
O(7)-Cu(3)-O(2)	85.6(2)
O(11)-Cu(3)-O(2)	101.3(3)
O(7)-Cu(3)-O(6)	90.3(2)
O(11)-Cu(3)-O(6)	83.7(2)
O(2)-Cu(3)-O(6)	169.3(3)
O(7)-Cu(3)-O(10)	85.5(2)
O(11)-Cu(3)-O(10)	89.8(2)
O(2)-Cu(3)-O(10)	85.6(3)
O(6)-Cu(3)-O(10)	104.0(2)
O(7)-Cu(3)-O(3)	102.1(3)
O(11)-Cu(3)-O(3)	83.4(2)
O(2)-Cu(3)-O(3)	88.7(2)
O(6)-Cu(3)-O(3)	82.5(2)
O(10)-Cu(3)-O(3)	170.1(3)
O(8)-Cu(4)-N(7)	95.3(4)
O(8)-Cu(4)-O(13)	95.9(3)
N(7)-Cu(4)-O(13)	156.3(3)
O(8)-Cu(4)-O(7)	172.8(3)
N(7)-Cu(4)-O(7)	84.6(3)
O(13)-Cu(4)-O(7)	86.8(3)
O(8)-Cu(4)-O(2)	95.0(3)
N(7)-Cu(4)-O(2)	123.0(3)
O(13)-Cu(4)-O(2)	76.7(3)
O(7)-Cu(4)-O(2)	79.1(3)
O(1)-Cu(5)-N(2)	95.3(4)
O(1)-Cu(5)-O(13)	96.5(3)
N(2)-Cu(5)-O(13)	157.9(4)
O(1)-Cu(5)-O(2)	174.7(3)
N(2)-Cu(5)-O(2)	83.2(4)
O(13)-Cu(5)-O(2)	86.6(3)
O(1)-Cu(5)-O(10)	96.5(3)
N(2)-Cu(5)-O(10)	122.6(3)
O(13)-Cu(5)-O(10)	74.4(2)
O(2)-Cu(5)-O(10)	80.1(2)
O(5)-Cu(6)-N(6)	96.1(4)
O(5)-Cu(6)-O(6)	174.9(3)
N(6)-Cu(6)-O(6)	85.4(3)
O(5)-Cu(6)-O(14)	94.5(3)
N(6)-Cu(6)-O(14)	151.3(3)
O(6)-Cu(6)-O(14)	86.4(3)
O(5)-Cu(6)-O(11)	96.0(3)
N(6)-Cu(6)-O(11)	128.9(3)
O(6)-Cu(6)-O(11)	79.3(2)

O(14)-Cu(6)-O(11)	76.1(2)
O(12)-Cu(7)-N(11)	94.0(3)
O(12)-Cu(7)-O(11)	177.0(3)
N(11)-Cu(7)-O(11)	85.5(3)
O(12)-Cu(7)-O(14)	96.0(3)
N(11)-Cu(7)-O(14)	152.6(3)
O(11)-Cu(7)-O(14)	85.7(3)
O(12)-Cu(7)-O(3)	98.3(3)
N(11)-Cu(7)-O(3)	128.6(3)
O(11)-Cu(7)-O(3)	79.8(3)
O(14)-Cu(7)-O(3)	74.9(2)

5.2. ***Table S4*.** Selected Bond lengths [Å] and angles [°] for **2**.

Cu(1)-O(3)	1.888(4)
Cu(1)-N(2)	1.922(4)
Cu(1)-O(2)	2.004(4)
Cu(1)-O(1)	2.007(3)
Cu(1)-O(2)#1	2.376(4)
Cu(2)-O(2)#2	2.102(4)
Cu(2)-O(2)#3	2.102(4)
Cu(2)-O(2)#1	2.102(4)
Cu(2)-O(2)	2.102(4)
Cu(2)-O(2)#4	2.102(4)
Cu(2)-O(2)#5	2.102(4)
Cu(3)-O(10)#6	1.912(10)
Cu(3)-O(7)	1.944(14)
Cu(3)-N(1)	1.978(3)
Cu(3)-O(6)	1.988(4)
Cu(3)-O(4)#7	2.167(8)
Cu(3)-O(4)	2.204(8)
O(3)-Cu(1)-N(2)	95.04(18)
O(3)-Cu(1)-O(2)	173.78(18)
N(2)-Cu(1)-O(2)	83.72(17)
O(3)-Cu(1)-O(1)	98.85(16)
N(2)-Cu(1)-O(1)	153.70(15)
O(2)-Cu(1)-O(1)	84.67(16)
O(3)-Cu(1)-O(2)#1	94.48(16)
N(2)-Cu(1)-O(2)#1	125.56(17)
O(2)-Cu(1)-O(2)#1	81.39(19)
O(1)-Cu(1)-O(2)#1	75.57(15)
O(2)#2-Cu(2)-O(2)#3	169.85(18)
O(2)#2-Cu(2)-O(2)#1	86.12(14)
O(2)#3-Cu(2)-O(2)#1	87.45(18)
O(2)#2-Cu(2)-O(2)	86.12(14)
O(2)#3-Cu(2)-O(2)	101.27(19)
O(2)#1-Cu(2)-O(2)	86.12(14)

O(2)#2-Cu(2)-O(2)#4	101.27(19)
O(2)#3-Cu(2)-O(2)#4	86.12(14)
O(2)#1-Cu(2)-O(2)#4	169.85(18)
O(2)-Cu(2)-O(2)#4	87.45(18)
O(2)#2-Cu(2)-O(2)#5	87.45(18)
O(2)#3-Cu(2)-O(2)#5	86.12(14)
O(2)#1-Cu(2)-O(2)#5	101.27(19)
O(2)-Cu(2)-O(2)#5	169.85(18)
O(2)#4-Cu(2)-O(2)#5	86.12(14)
O(10)#6-Cu(3)-O(7)	69.1(6)
O(10)#6-Cu(3)-N(1)	83.7(4)
O(7)-Cu(3)-N(1)	87.0(5)
O(10)#6-Cu(3)-O(6)	100.0(6)
O(7)-Cu(3)-O(6)	95.3(6)
N(1)-Cu(3)-O(6)	176.2(6)
O(10)#6-Cu(3)-O(4)#7	108.3(3)
O(7)-Cu(3)-O(4)#7	175.6(5)
N(1)-Cu(3)-O(4)#7	89.2(2)
O(6)-Cu(3)-O(4)#7	88.7(4)
O(10)#6-Cu(3)-O(4)	166.6(3)
O(7)-Cu(3)-O(4)	100.5(5)
N(1)-Cu(3)-O(4)	87.5(2)
O(6)-Cu(3)-O(4)	89.1(5)
O(4)#7-Cu(3)-O(4)	81.6(2)

Symmetry transformations used to generate equivalent atoms:

#1 -y,x-y,z #2 -x+y,-x,z #3 x-y,-y,-z+1
#4 -x,-x+y,-z+1 #5 y,x,-z+1 #6 -y+1,x-y,z
#7 x,x-y,-z+1/2 #8 -x+y+1,-x+1,z
#9 -y+1,-x+1,-z+1/2

5.3. *Table S5*. Selected Bond lengths [Å] and angles [°] for **3.**

Cu(1)-O(5)	2.091(5)
Cu(1)-O(5)#1	2.091(5)
Cu(1)-O(5)#2	2.091(5)
Cu(1)-O(4)#1	2.111(5)
Cu(1)-O(4)	2.111(5)
Cu(1)-O(4)#2	2.111(5)
Cu(2)-O(3)	1.867(5)
Cu(2)-N(2)	1.901(6)
Cu(2)-O(4)	1.975(5)
Cu(2)-O(1)	1.992(4)
Cu(2)-O(4)#2	2.381(5)
Cu(3)-O(6)	1.877(6)
Cu(3)-N(3)	1.898(7)
Cu(3)-O(5)	1.958(5)

Cu(3)-O(2)	1.971(4)
Cu(3)-O(5)#1	2.404(5)
Cu(4)-O(8)	1.782(18)
Cu(4)-O(11)	1.894(15)
Cu(4)-N(4)	1.952(7)
Cu(4)-O(10)	1.977(7)
Cu(4)-O(14)#3	2.093(13)
Cu(5)-O(7A)#4	1.89(2)
Cu(5)-O(7A)	1.89(2)
Cu(5)-N(1)	1.957(3)
Cu(5)-N(1)#4	1.957(3)
Cu(5)-O(15)#4	2.158(15)
Cu(5)-O(15)	2.158(15)
Cu(5)-O(7)	2.17(3)
Cu(5)-O(7)#4	2.17(3)
O(5)-Cu(1)-O(5)#1	85.0(2)
O(5)-Cu(1)-O(5)#2	85.0(2)
O(5)#1-Cu(1)-O(5)#2	85.0(2)
O(5)-Cu(1)-O(4)#1	101.9(2)
O(5)#1-Cu(1)-O(4)#1	88.86(19)
O(5)#2-Cu(1)-O(4)#1	170.32(19)
O(5)-Cu(1)-O(4)	88.86(19)
O(5)#1-Cu(1)-O(4)	170.32(19)
O(5)#2-Cu(1)-O(4)	101.9(2)
O(4)#1-Cu(1)-O(4)	85.1(2)
O(5)-Cu(1)-O(4)#2	170.32(19)
O(5)#1-Cu(1)-O(4)#2	101.9(2)
O(5)#2-Cu(1)-O(4)#2	88.86(19)
O(4)#1-Cu(1)-O(4)#2	85.1(2)
O(4)-Cu(1)-O(4)#2	85.1(2)
O(3)-Cu(2)-N(2)	96.3(3)
O(3)-Cu(2)-O(4)	174.5(3)
N(2)-Cu(2)-O(4)	84.5(2)
O(3)-Cu(2)-O(1)	94.8(2)
N(2)-Cu(2)-O(1)	154.7(2)
O(4)-Cu(2)-O(1)	86.7(2)
O(3)-Cu(2)-O(4)#2	93.8(2)
N(2)-Cu(2)-O(4)#2	125.3(2)
O(4)-Cu(2)-O(4)#2	81.3(3)
O(1)-Cu(2)-O(4)#2	76.4(2)
O(6)-Cu(3)-N(3)	96.2(3)
O(6)-Cu(3)-O(5)	174.6(3)
N(3)-Cu(3)-O(5)	85.0(3)
O(6)-Cu(3)-O(2)	94.8(3)
N(3)-Cu(3)-O(2)	157.3(3)
O(5)-Cu(3)-O(2)	85.9(3)
O(6)-Cu(3)-O(5)#1	95.0(3)
N(3)-Cu(3)-O(5)#1	123.8(3)
O(5)-Cu(3)-O(5)#1	80.0(3)
O(2)-Cu(3)-O(5)#1	74.7(2)
O(8)-Cu(4)-O(11)	145.8(9)

O(8)-Cu(4)-N(4)	85.3(7)
O(11)-Cu(4)-N(4)	93.7(6)
O(8)-Cu(4)-O(10)	88.2(7)
O(11)-Cu(4)-O(10)	93.2(6)
N(4)-Cu(4)-O(10)	173.0(5)
O(8)-Cu(4)-O(14)#3	109.0(8)
O(11)-Cu(4)-O(14)#3	105.2(6)
N(4)-Cu(4)-O(14)#3	89.5(5)
O(10)-Cu(4)-O(14)#3	90.1(5)
O(7A)#4-Cu(5)-O(7A)	166.5(15)
O(7A)#4-Cu(5)-N(1)	91.8(7)
O(7A)-Cu(5)-N(1)	90.2(7)
O(7A)#4-Cu(5)-N(1)#4	90.2(7)
O(7A)-Cu(5)-N(1)#4	91.8(7)
N(1)-Cu(5)-N(1)#4	163.1(3)
O(7A)#4-Cu(5)-O(15)#4	64.9(8)
O(7A)-Cu(5)-O(15)#4	101.6(8)
N(1)-Cu(5)-O(15)#4	98.0(5)
N(1)#4-Cu(5)-O(15)#4	98.1(5)
O(7A)#4-Cu(5)-O(15)	101.6(8)
O(7A)-Cu(5)-O(15)	64.9(8)
N(1)-Cu(5)-O(15)	98.1(5)
N(1)#4-Cu(5)-O(15)	98.0(5)
O(15)#4-Cu(5)-O(15)	36.7(7)
O(7A)#4-Cu(5)-O(7)	167.1(14)
O(7A)-Cu(5)-O(7)	26.4(9)
N(1)-Cu(5)-O(7)	87.0(8)
N(1)#4-Cu(5)-O(7)	87.3(8)
O(15)#4-Cu(5)-O(7)	128.0(10)
O(15)-Cu(5)-O(7)	91.3(10)
O(7A)#4-Cu(5)-O(7)#4	26.4(9)
O(7A)-Cu(5)-O(7)#4	167.1(14)
N(1)-Cu(5)-O(7)#4	87.3(8)
N(1)#4-Cu(5)-O(7)#4	87.0(8)
O(15)#4-Cu(5)-O(7)#4	91.3(10)
O(15)-Cu(5)-O(7)#4	128.0(10)
O(7)-Cu(5)-O(7)#4	140.7(18))

Symmetry transformations used to generate equivalent atoms:

#1 -y+1,x-y+1,z #2 -x+y,-x+1,z #3 -y+1,x-y+2,z
#4 -x+y+1,y,-z+1/2 #5 -x+y-1,-x+1,z
#6 y-1,x+1,-z+1 #7 x,x-y,-z+1/2 #8 -x+y+1,-x+1,z
#9 -y+1,-x+1,-z+1/2 #10 -y+1,x-y,z

6.1. ***Figure S1.*** View of the triple-stranded heptametallic helicate (a) and the heptanuclear core (b) in **1.**

(a)

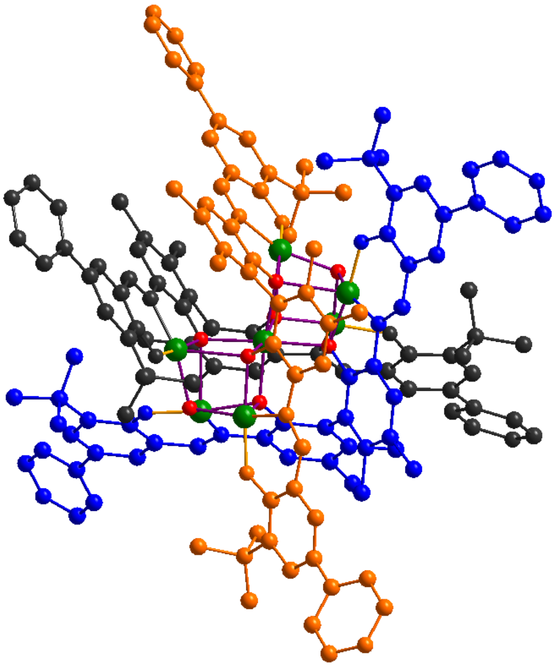

(b)

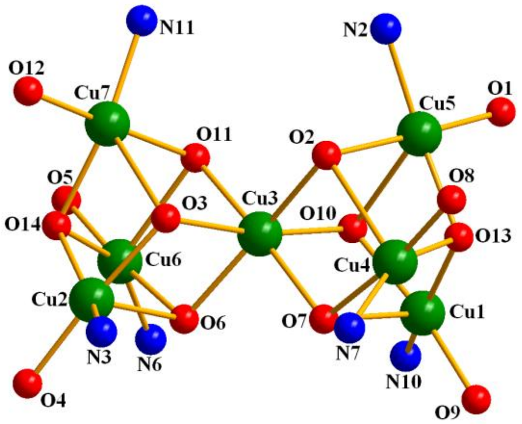

6.2. ***Figure S2.*** A view of the packing of **1** along the c-axis

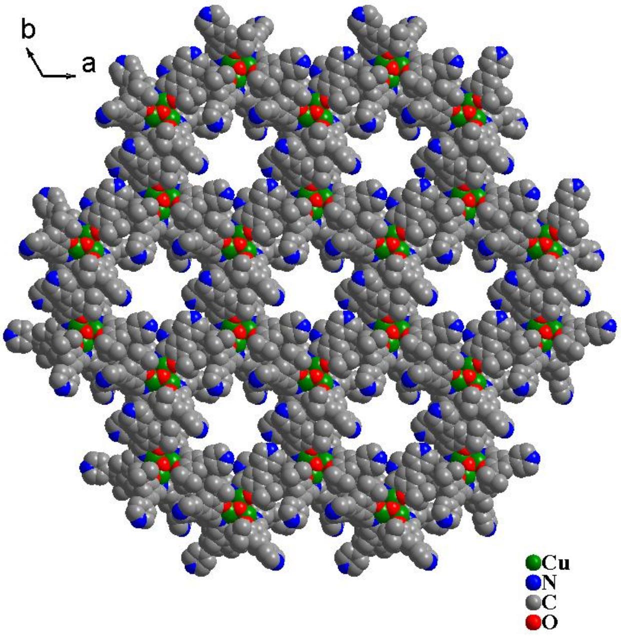

6.3. *Figure S3*. Intermolecular CH ··π and π ··π interactions between Cu_7 helicates in complex **1**

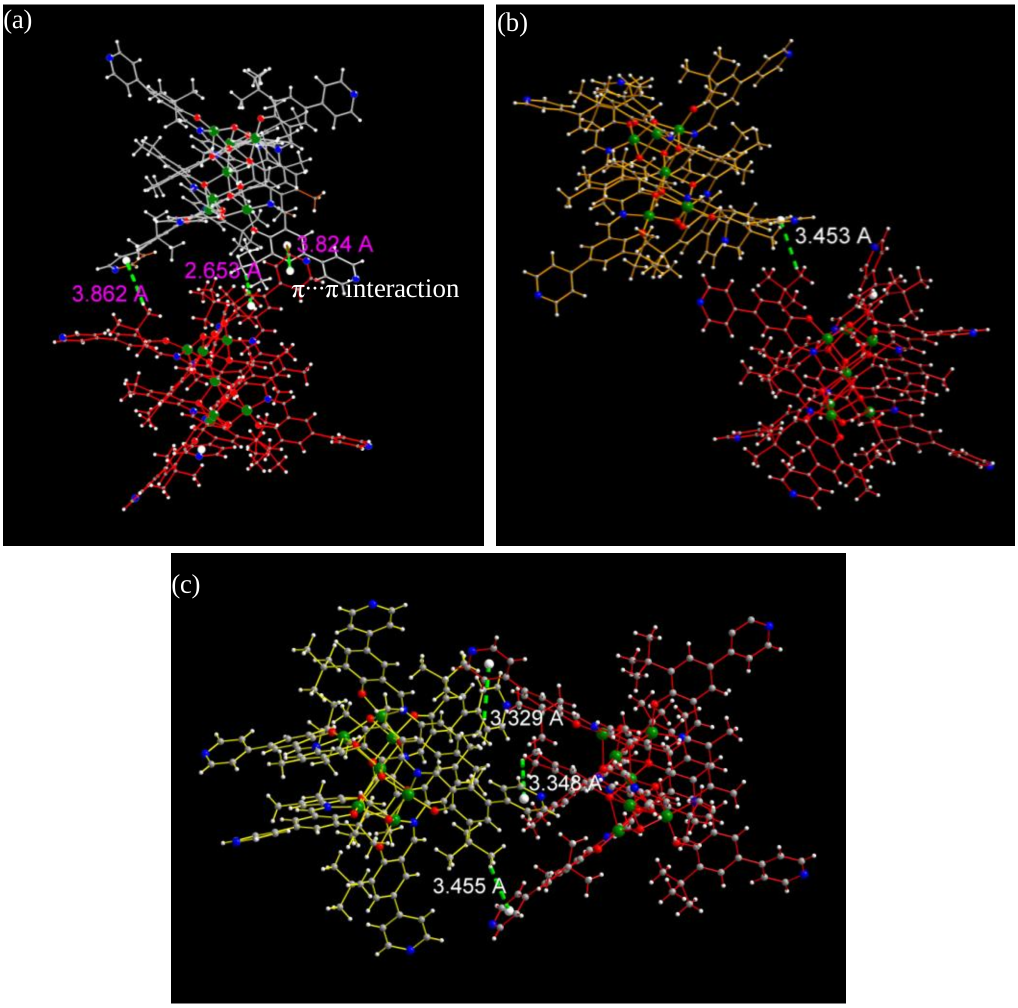

6.4. *Figure S4*. The space filling mode of **1**

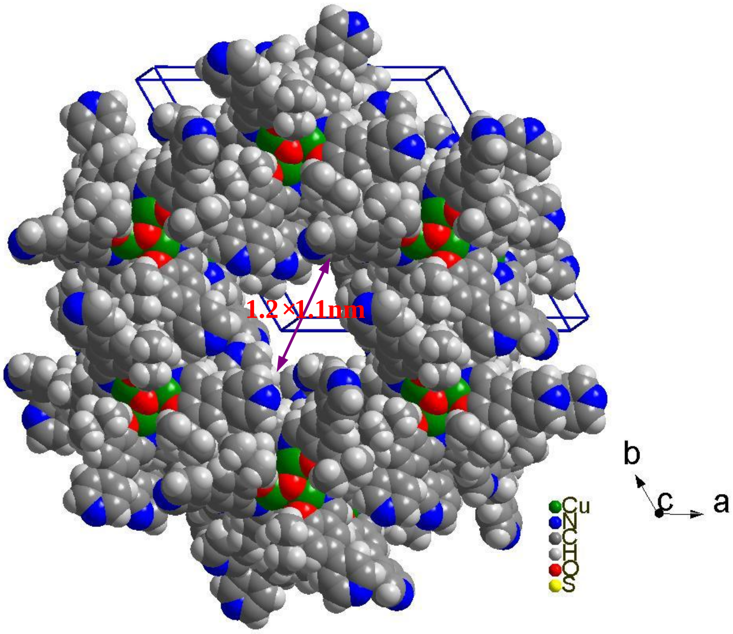

7.1. ***Figure S5*** A view of the asymmetric unit in **2** (the guest molecules were not included).

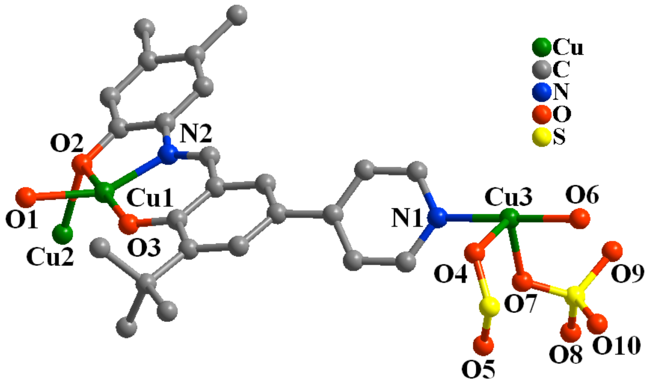

7.2. ***Figure S6***. View of the hexanuclear copper cluster (Cu_6-α) (a) and its coordination environment (b) in **2** (each pyridine unit stands for one Cu_7 helicate in the upper figure)

(a)

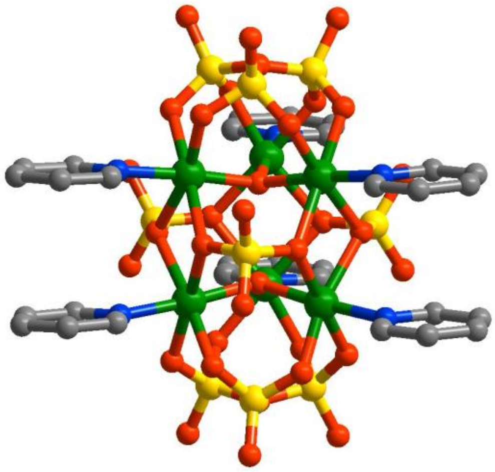

(b)

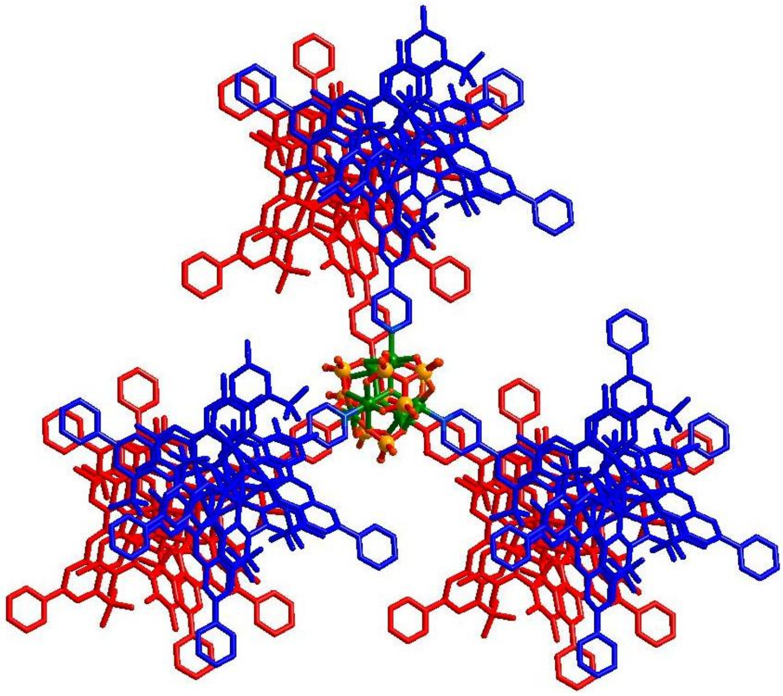

7.3. *Figure S7*. Coordination environments of the Cu_7 helicate in **2**.

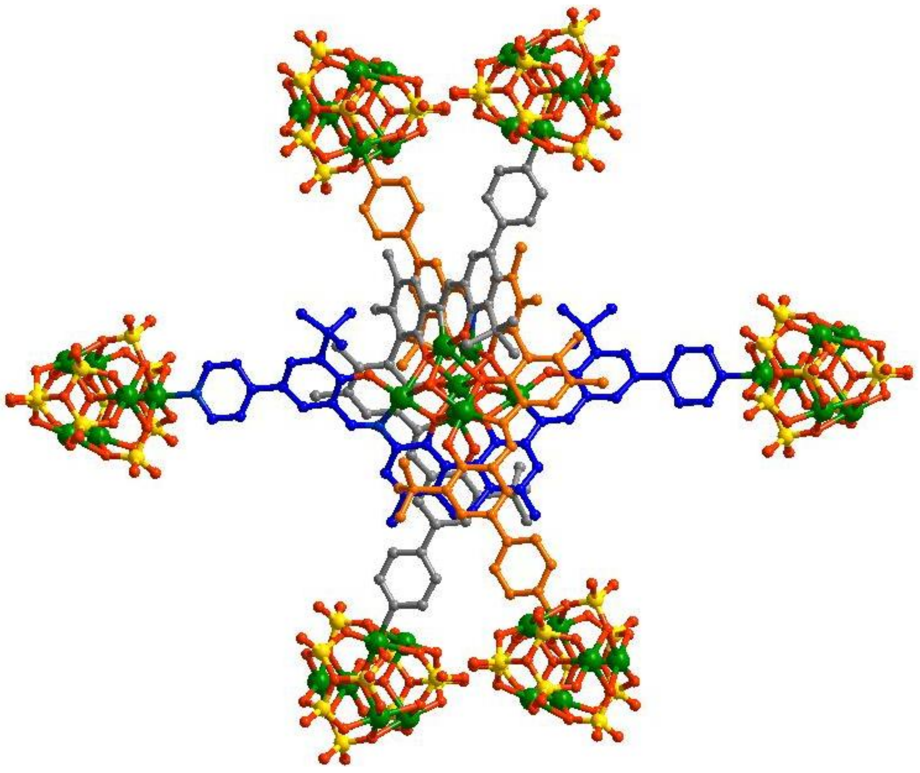

7.4. *Figure S8*. The $4^6 3^6$ cage in compound **2** (red point: the Cu_6-α cluster; the green point: a Cu_7 helicate).

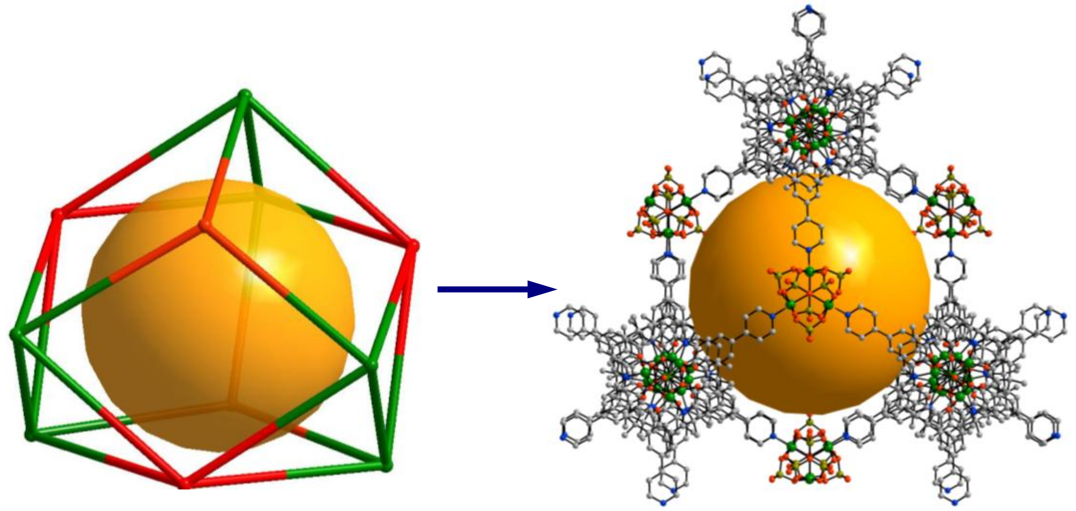

7.5. ***Figure S9.*** A view of 3D structures of **2**.

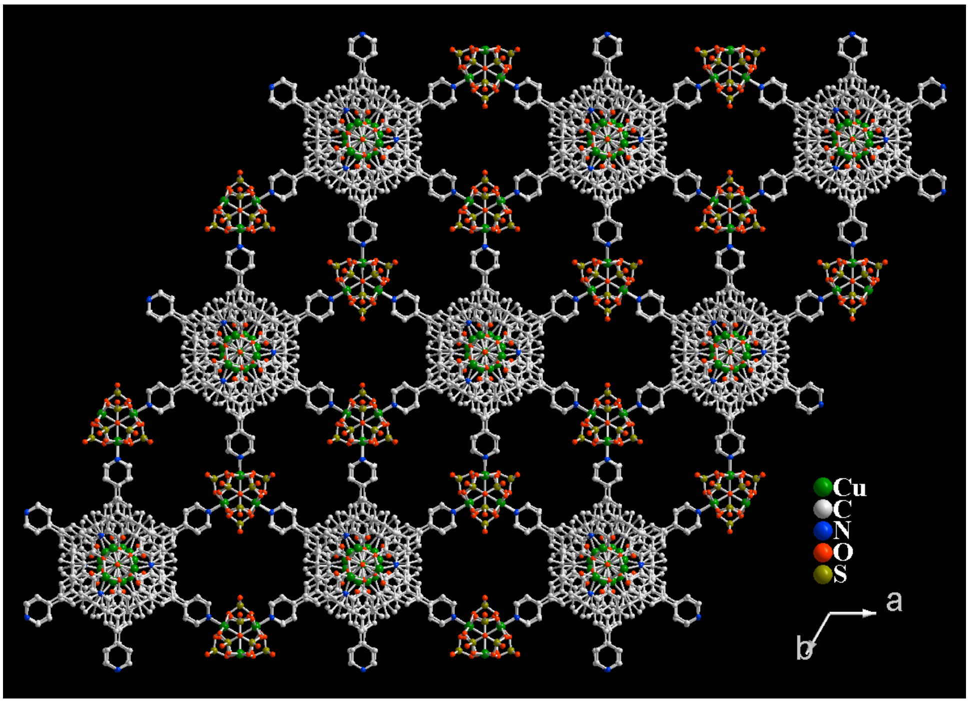

8.1. ***Figure S10.*** A view of the asymmetric unit in **3** (the guest molecules were not included).

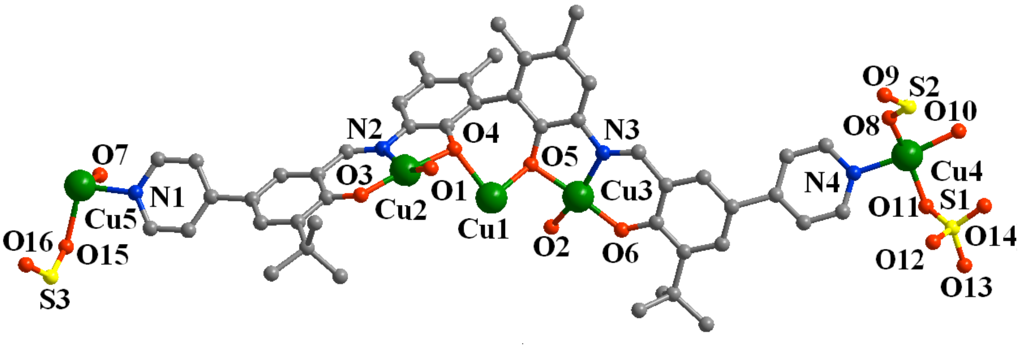

8.2. ***Figure S11.*** (a) The hexanuclear copper cluster (Cu_6-α) and (b) the trinuclear copper cluster $[Cu_3(SO_4)(H_2O)_6]$ in **3.**

(a)

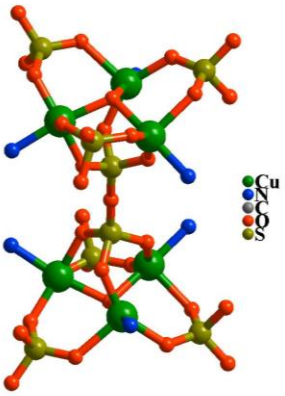

(b)

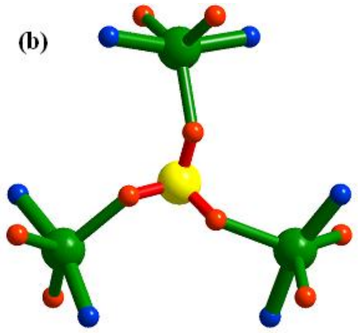

8.3. ***Figure S12*****.** The coordination environments of the hexanuclear Cu cluster (a), the trinuclear Cu cluster (b) and the Cu_7 helciate (c) in **2.**

(a)

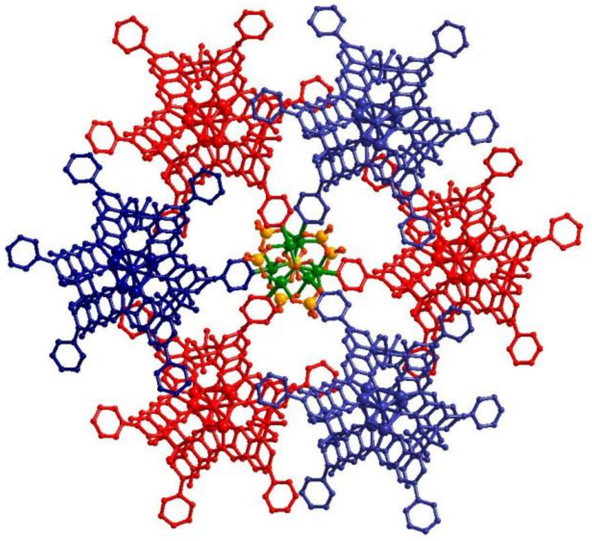

(b)

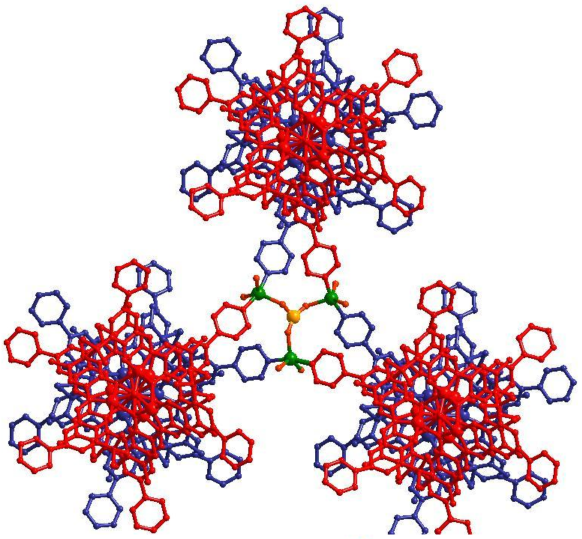

(c)

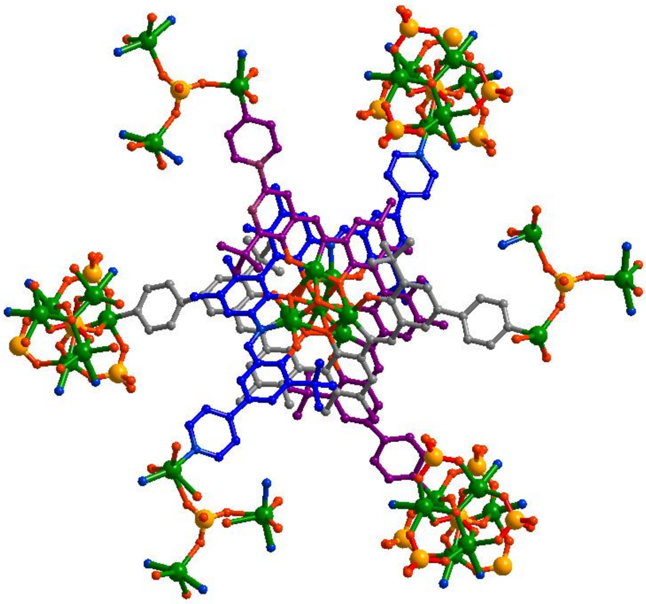

8.4. ***Figure S13.*** The $4^6 3^6$ (a) and 4^6 (b)cages in **3** (red point: the Cu_6-α cluster; the green point: a Cu_7 helicate; the purple point: the Cu_3 cluster)

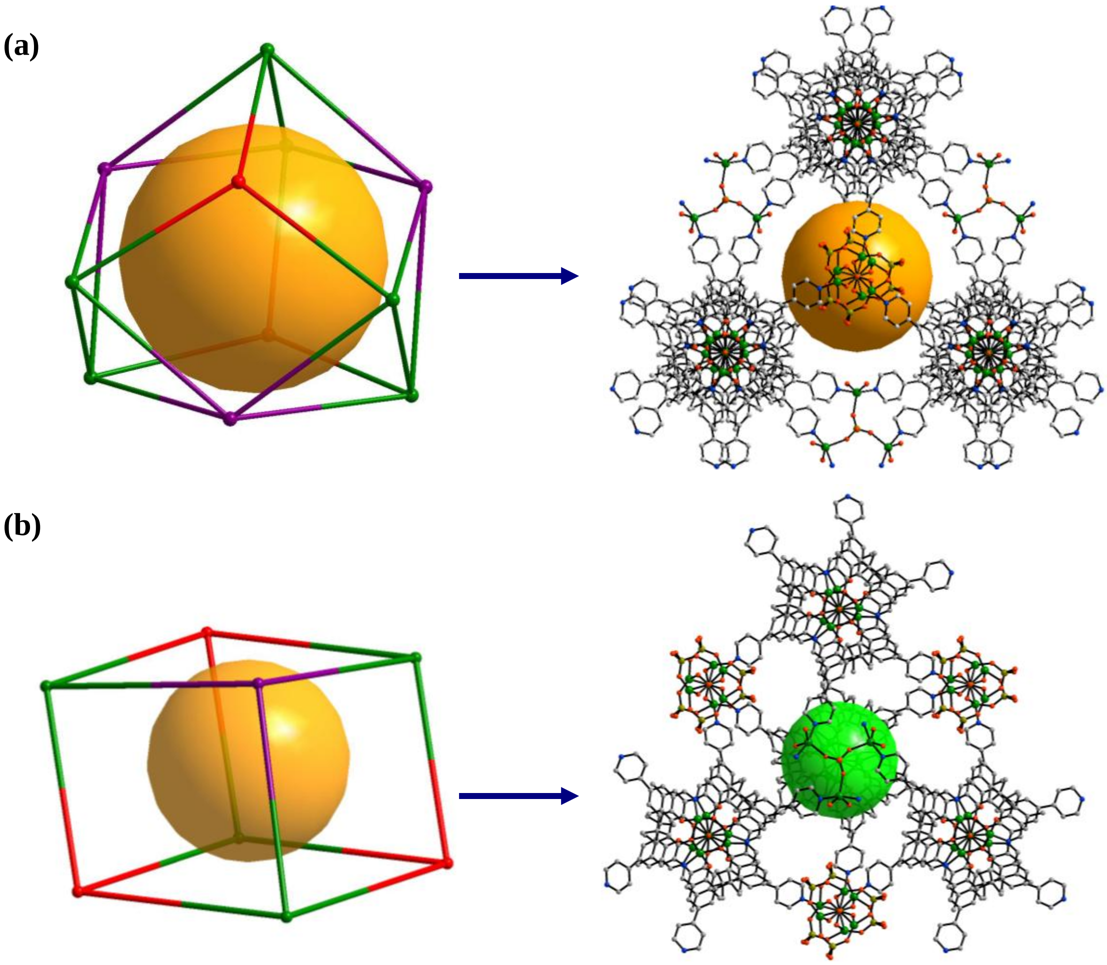

8.5. ***Figure S14.*** A view of 3D structure of **3.**

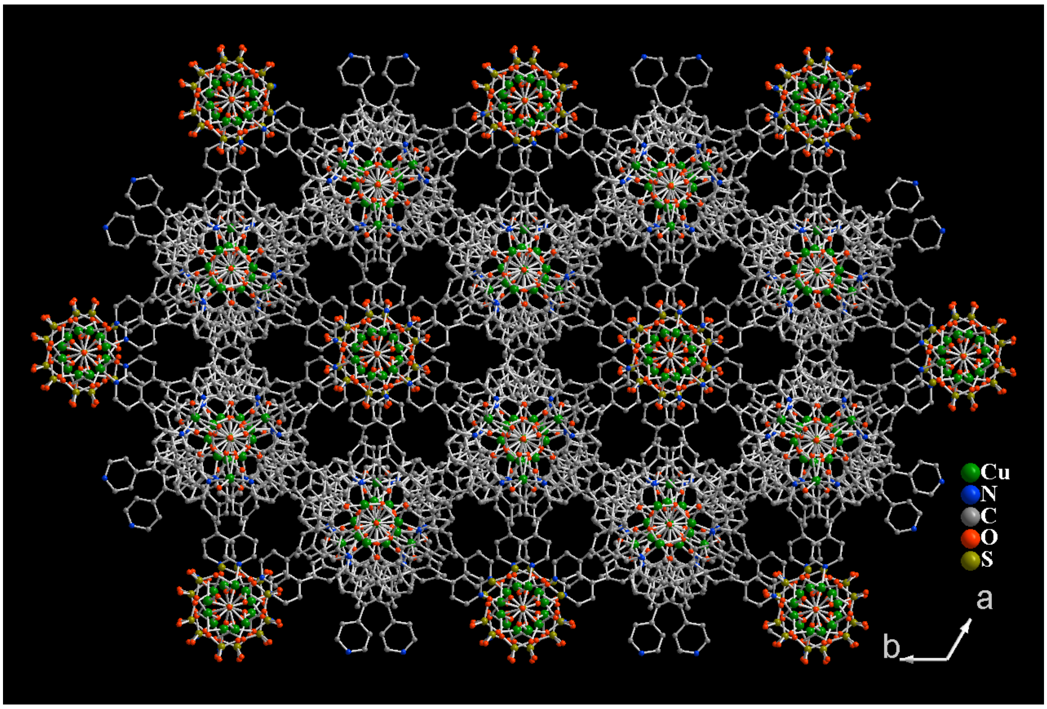

9.1. ***Figure S15***. The PXRD patterns of **1** and the removed solvent PXRD of **1** and the simulated PXRD pattern of **1**.

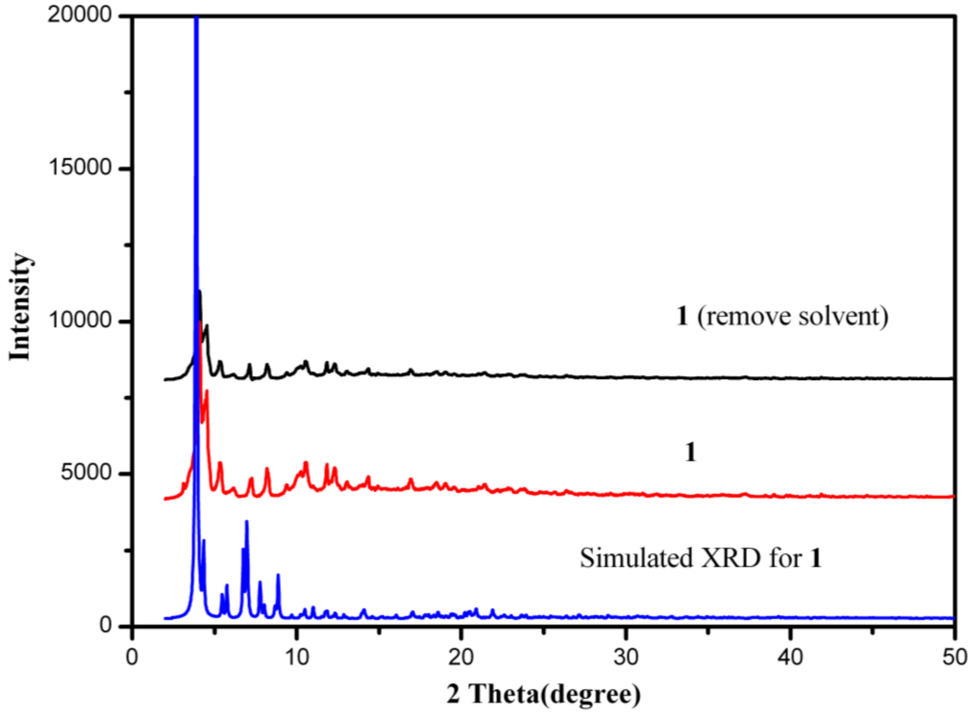

9.2. ***Figure S16***. The PXRD patterns of **2** and the removed solvent PXRD of **2** and the simulated PXRD pattern of **2**.

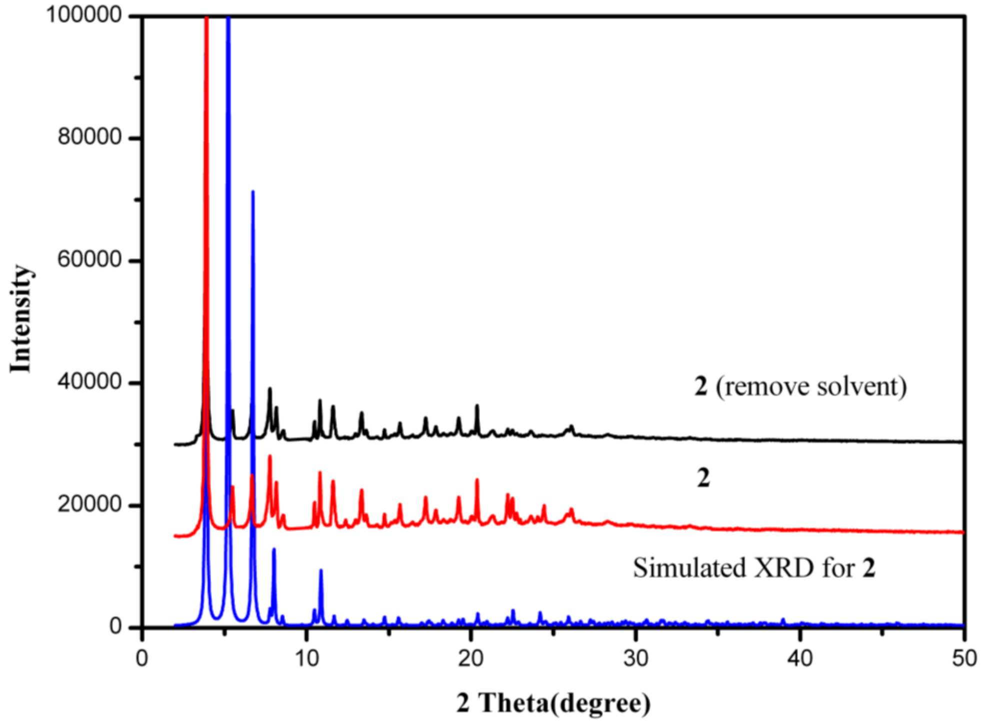

9.3. ***Figure S17***. The PXRD patterns of **3** and the removed solvent PXRD of **3** and the simulated PXRD pattern of **3**.

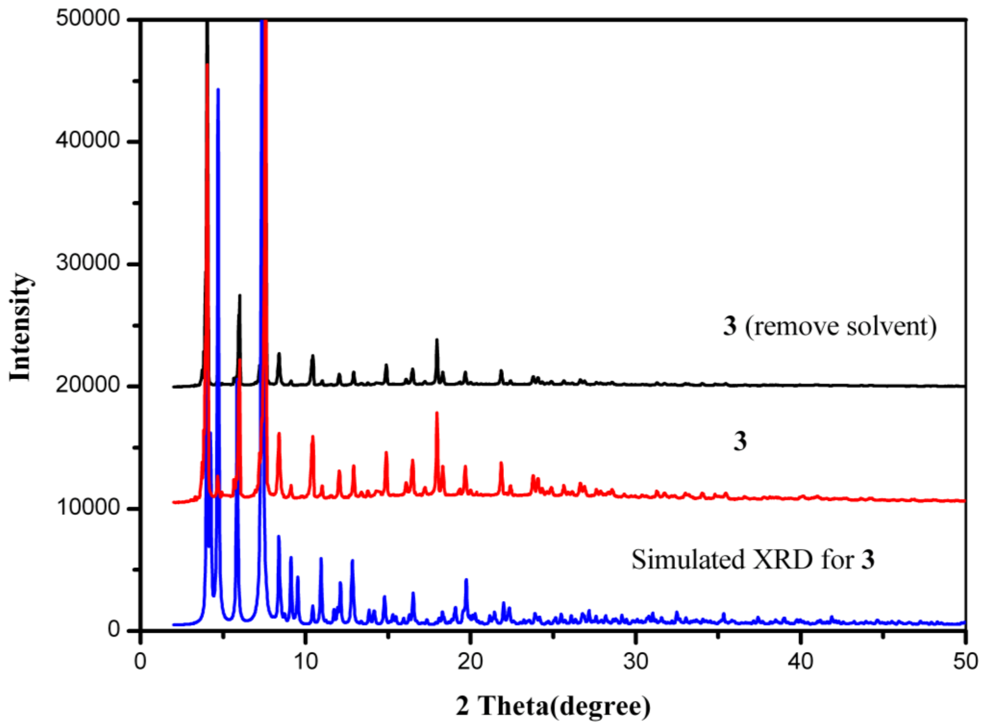

10. ***Figure S18***. TGA curves of **1, 2** and **3**

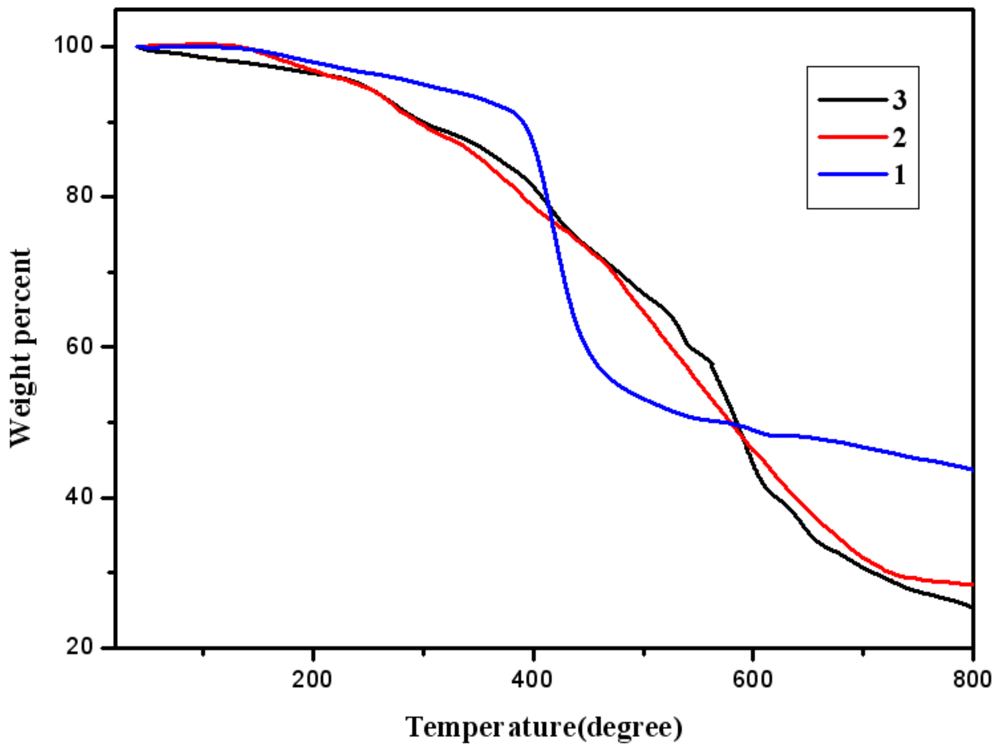

11.1 *Figure S19A*. Solid state CD spectra of (*R*)/(S)- **L**-(2MOM-2H), **-1, -2** and **-3**

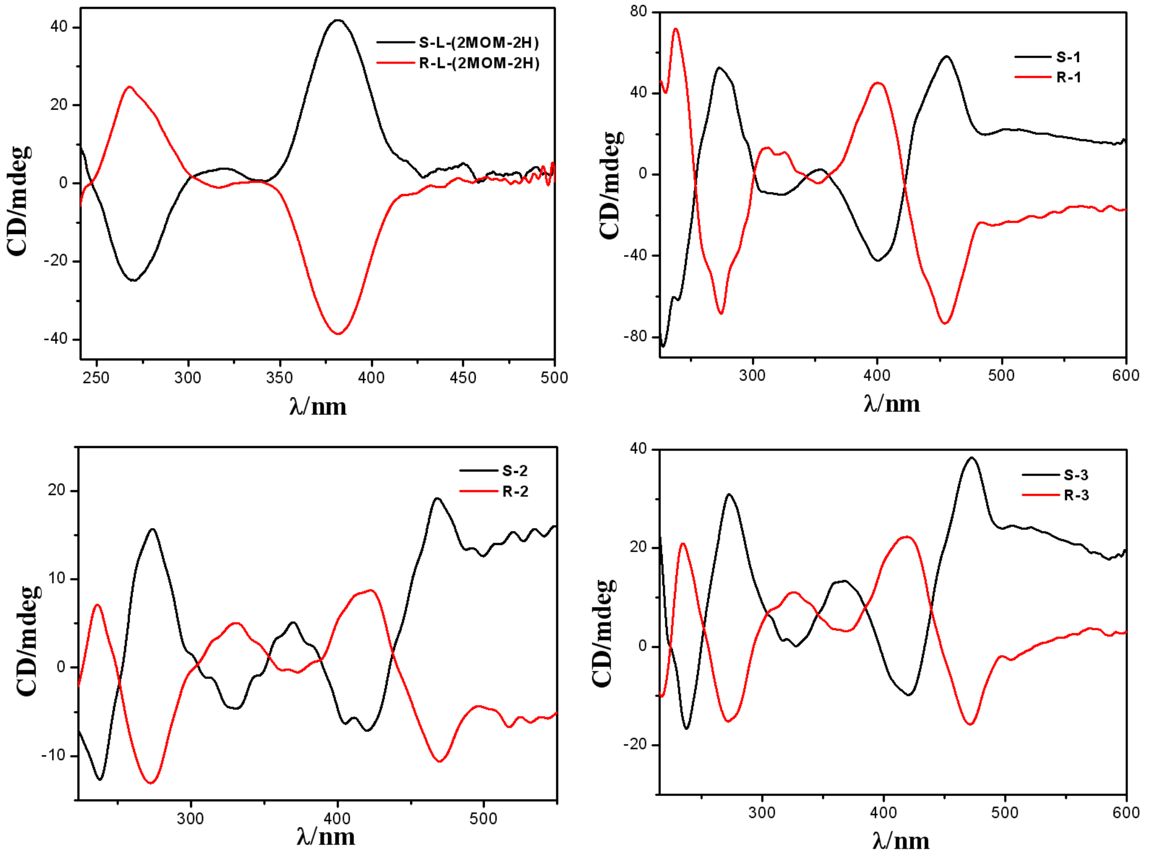

11.2 *Figure S19B*. CD spectra of (*R*)- **L**-(2MOM-2H) and (*S*)-**1** at different temperatures in DMSO. (The reversible and slight changes in CD spectra indicate the enantiopurity of the ligand and **1** in the range between 20 and 100 °C).

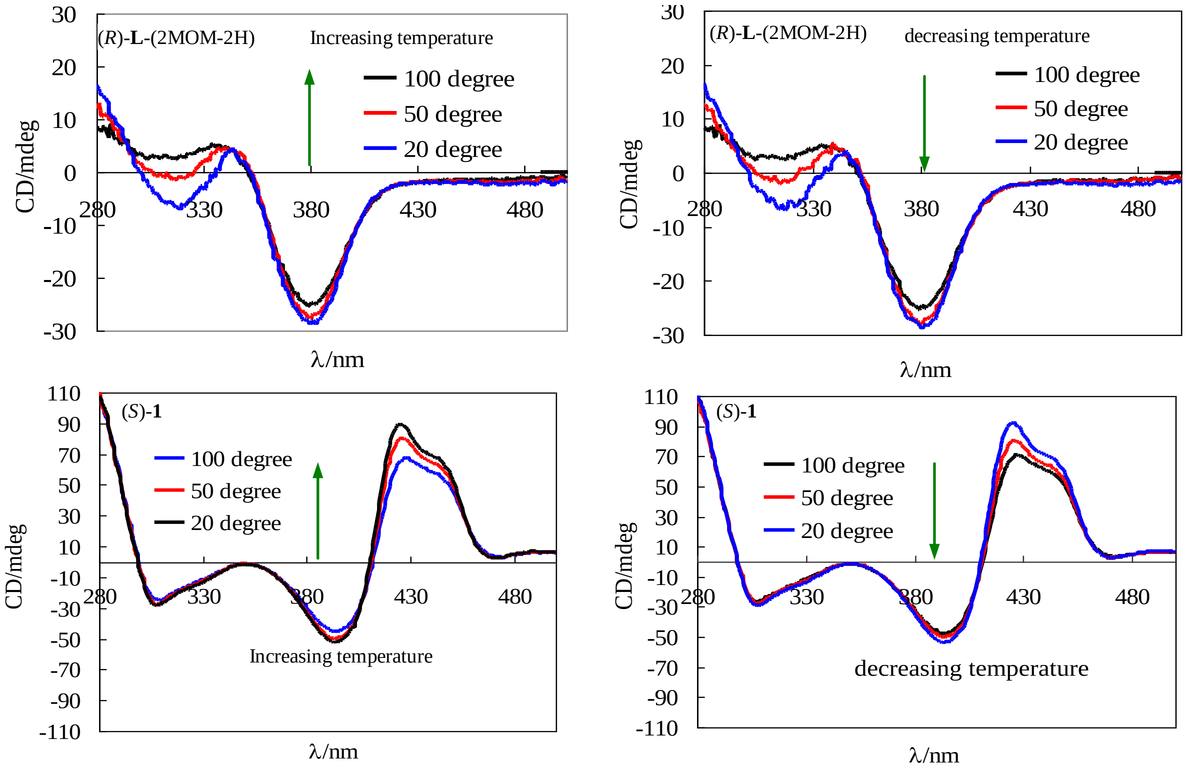

12. ***Figure S20***. UV/Vis absorption spectra of **1-3** and **L** in the solid state at room temperature.

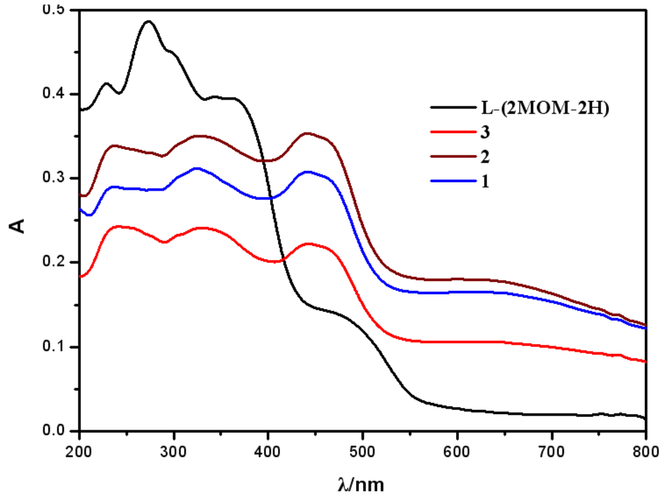

13. ***Figure S21***. UV/Vis absorption spectra of **1** at R.T. 80°C and 100°C in DMSO

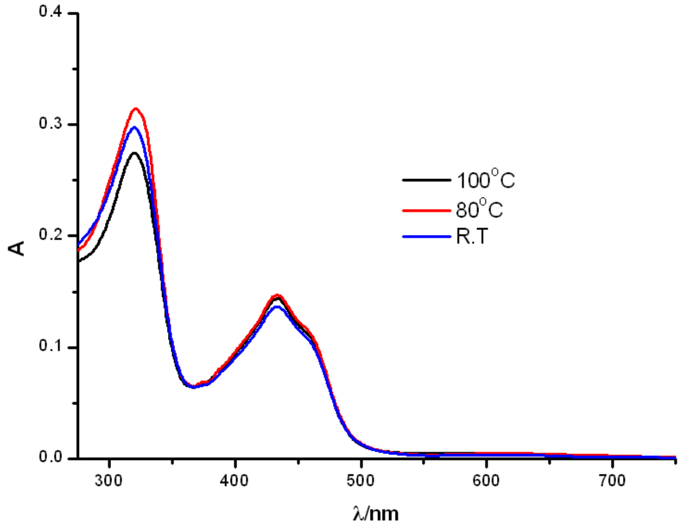

14.1 ***Figure S22.*** The N_2 adsorption isotherms for **1**

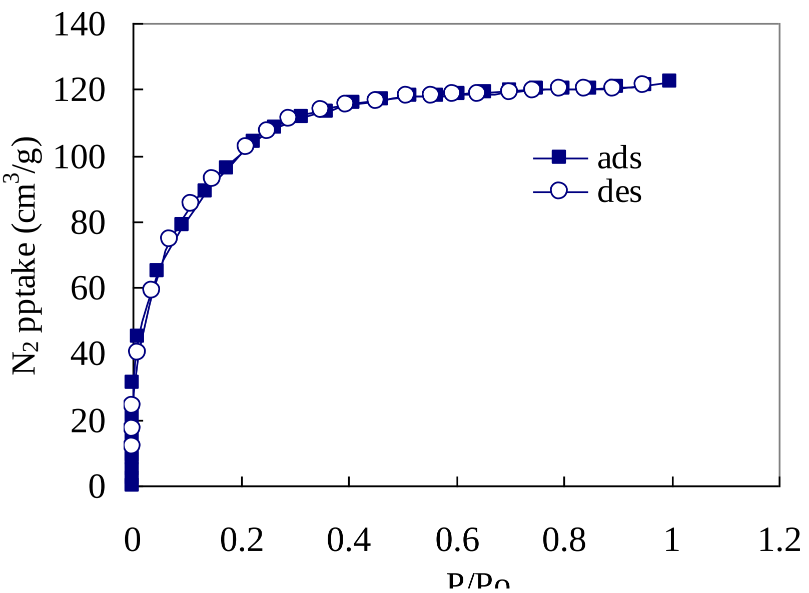

14.2 ***Figure S23.*** The N_2 adsorption isotherms for **2**

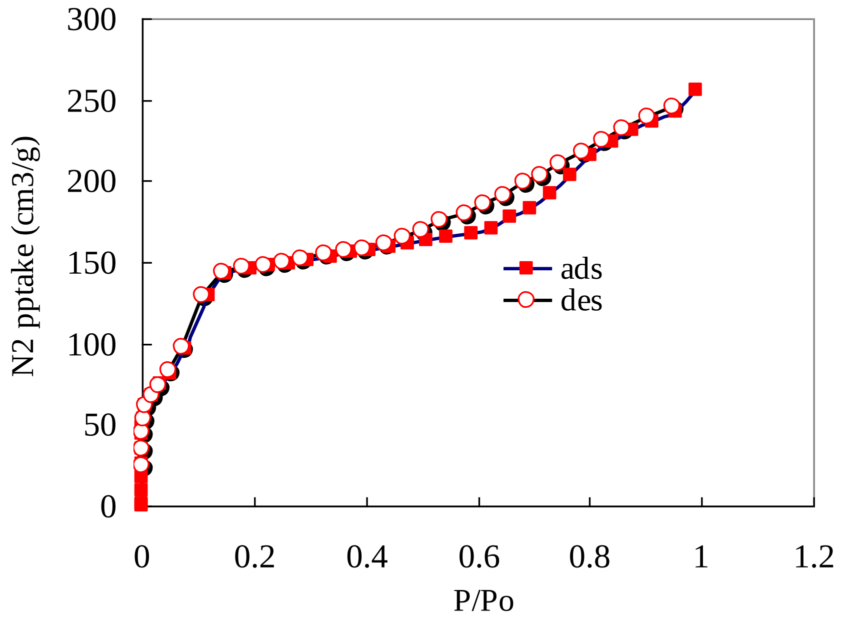

14.3 ***Figure S24*****.** The N_2 adsorption isotherms for **3**

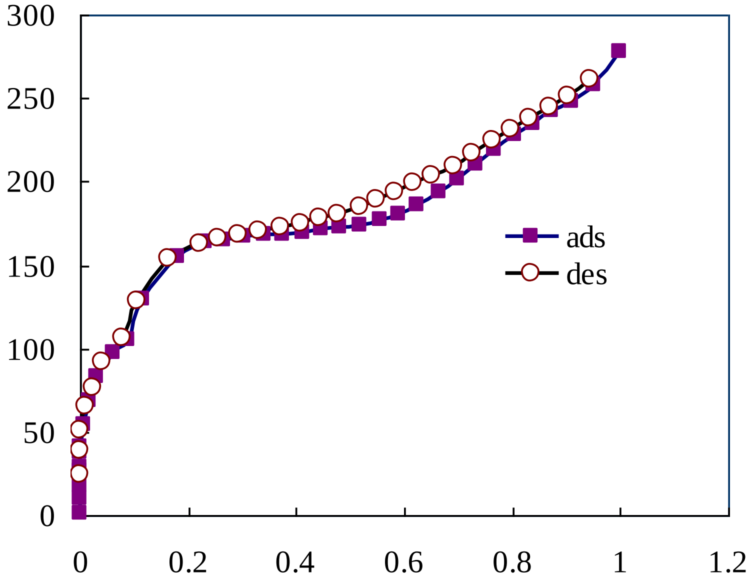

15.1 *Figure S25* Mass spectra of the ligand **L-**(2MOM-2H) and related organic compounds

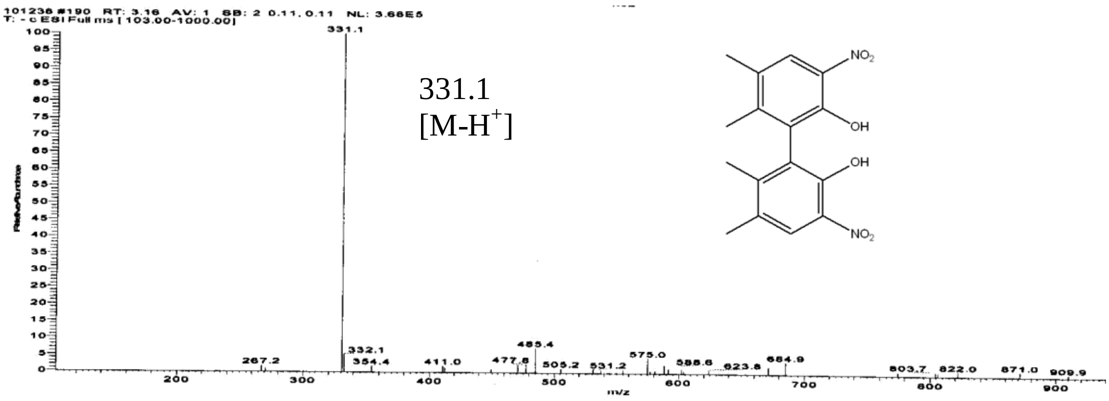

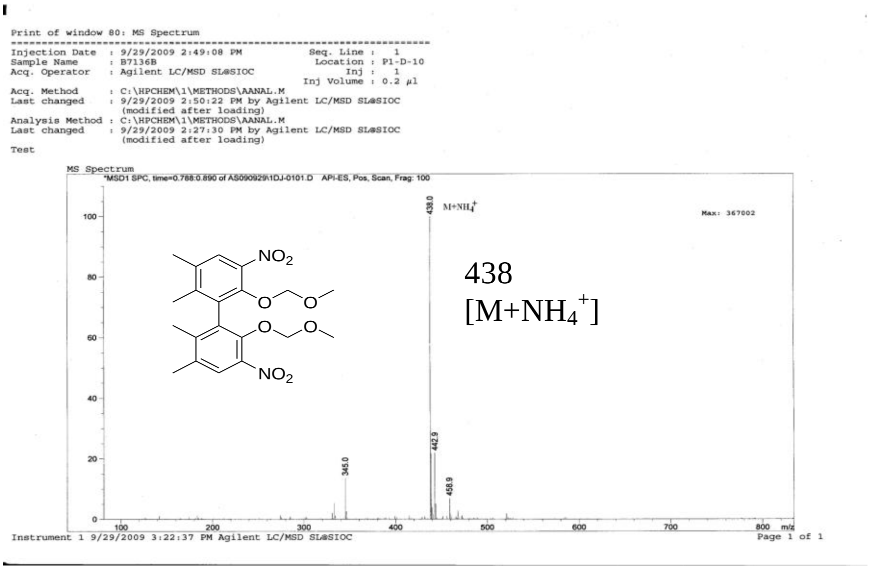

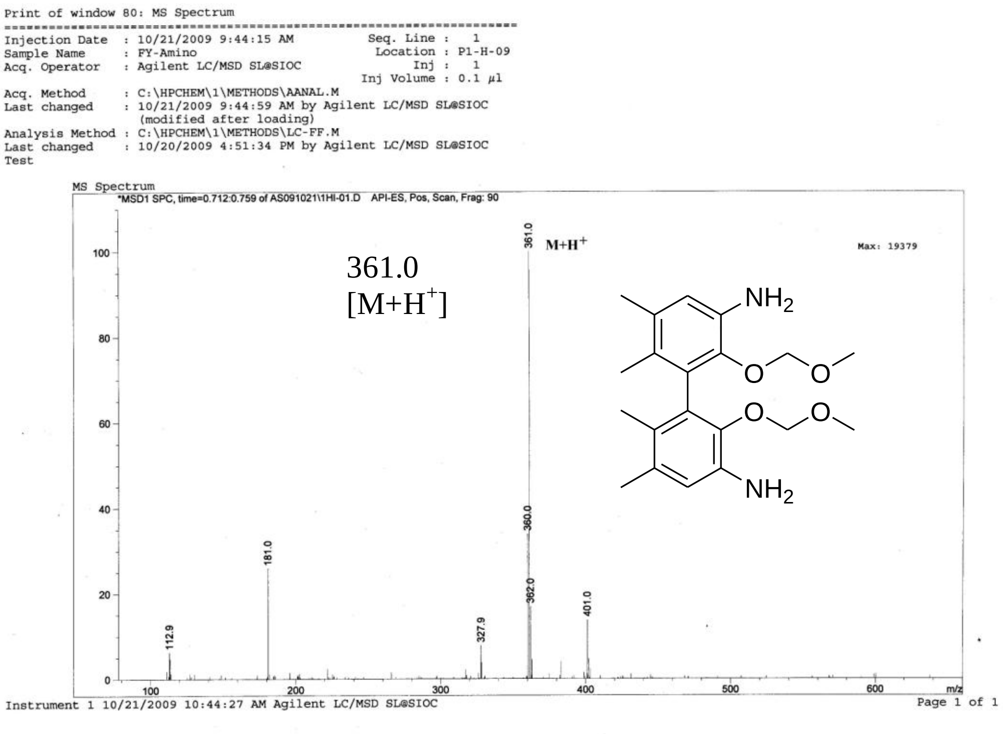

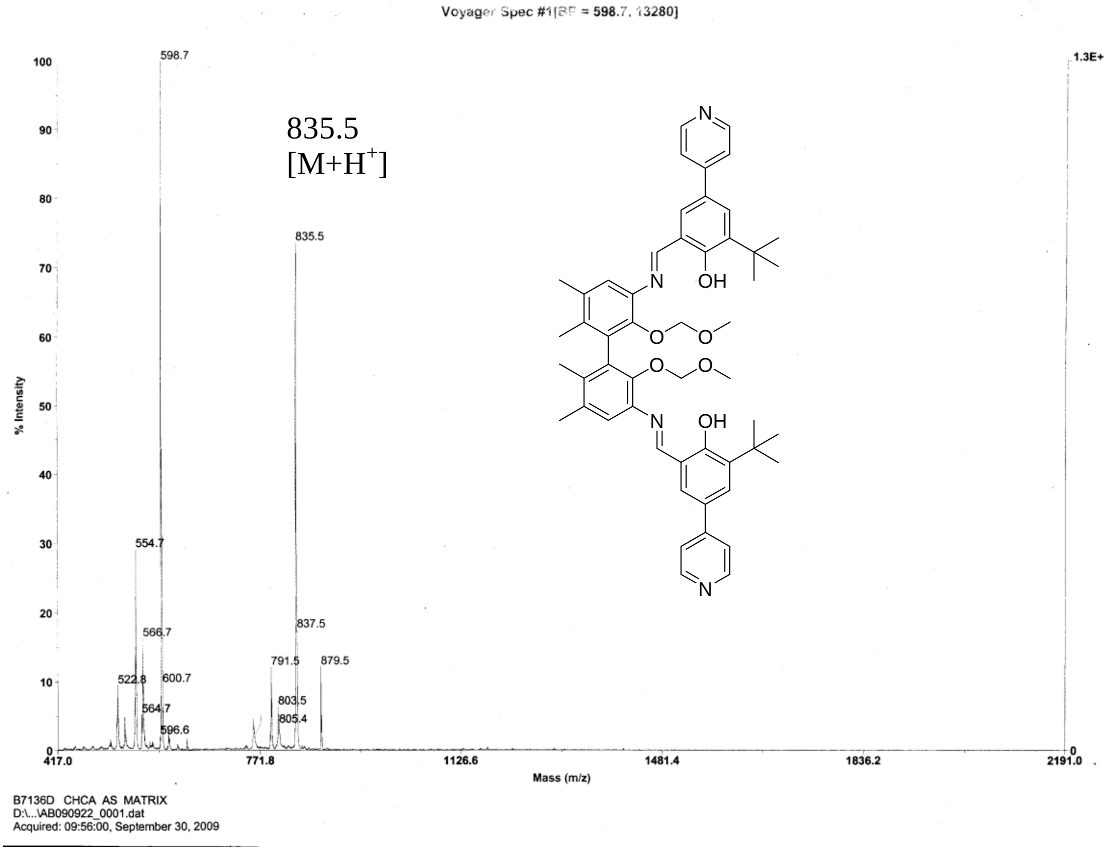

15.5 ***Figure 26*** ESI-Mass of the helicate **1**.

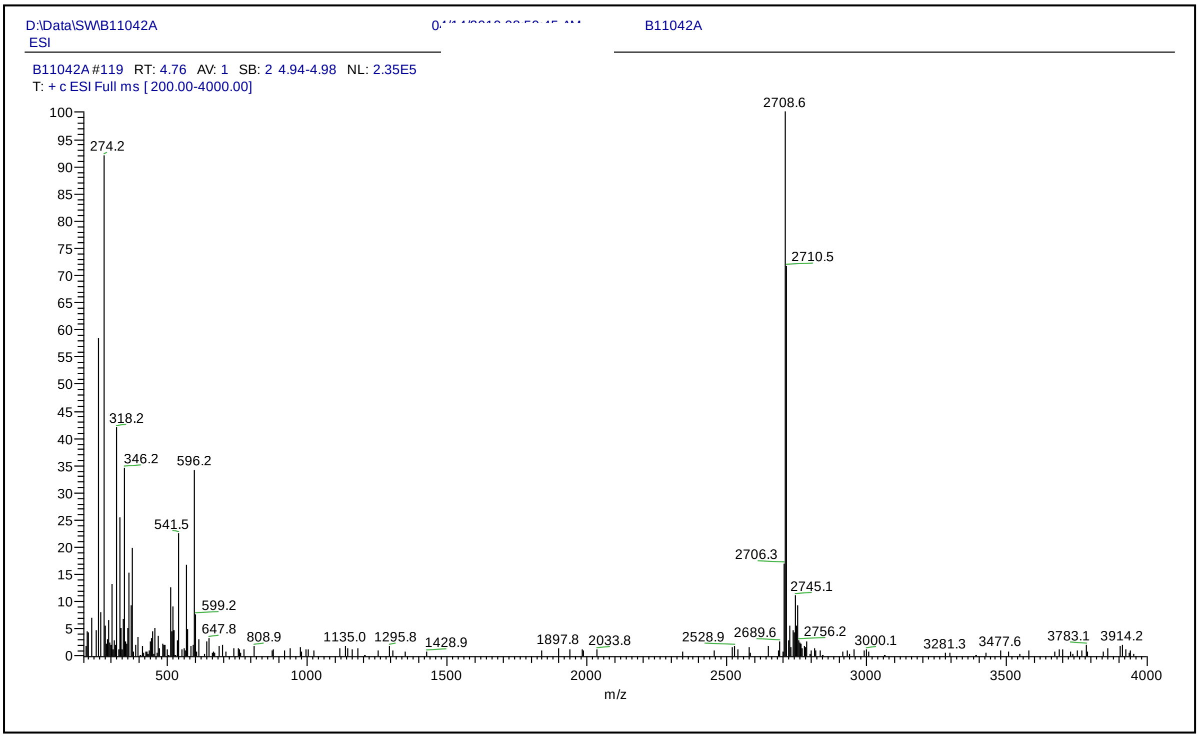

16.1. ***Figure S27*** ^{1}H and ^{13}C NMR of related organic compounds

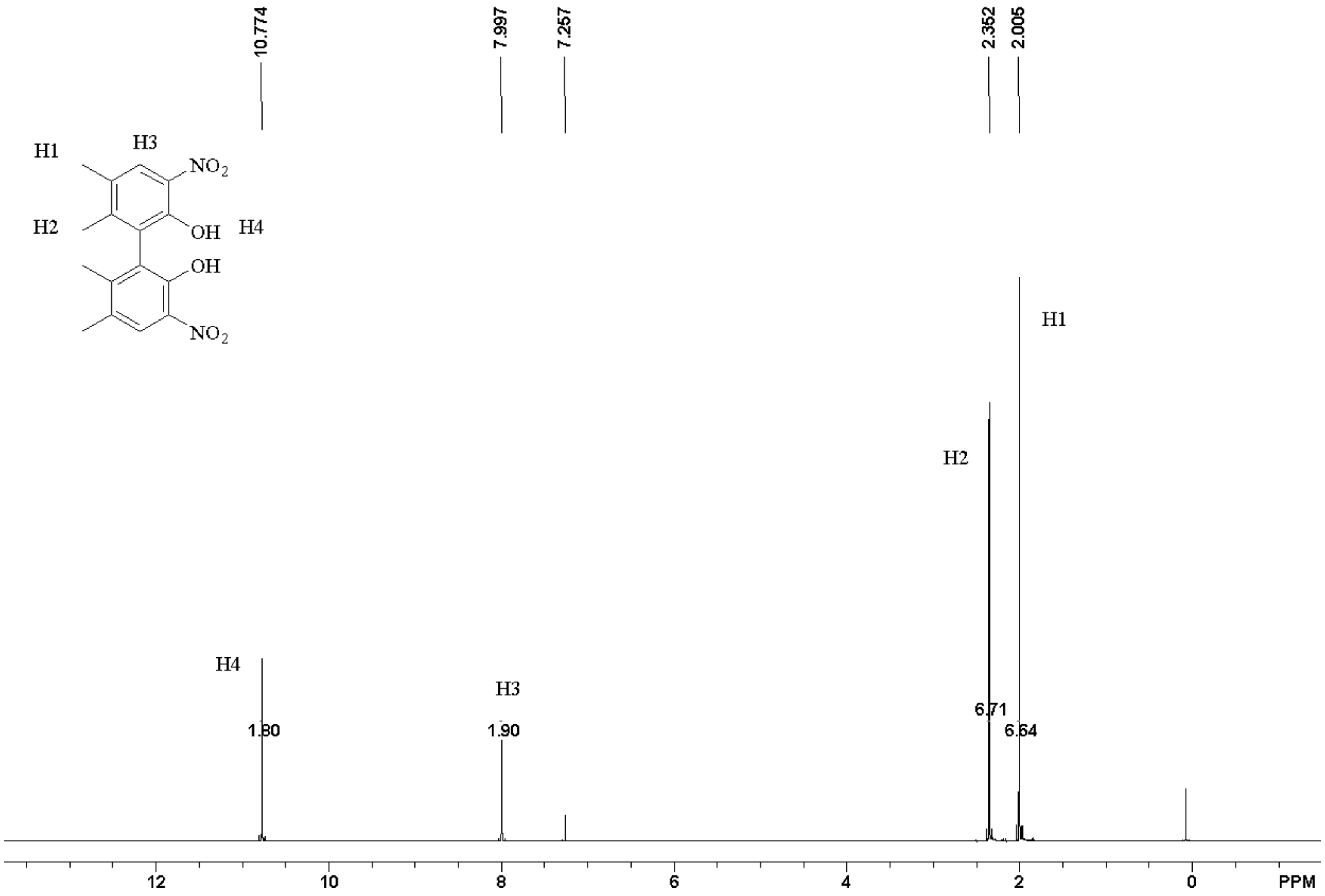

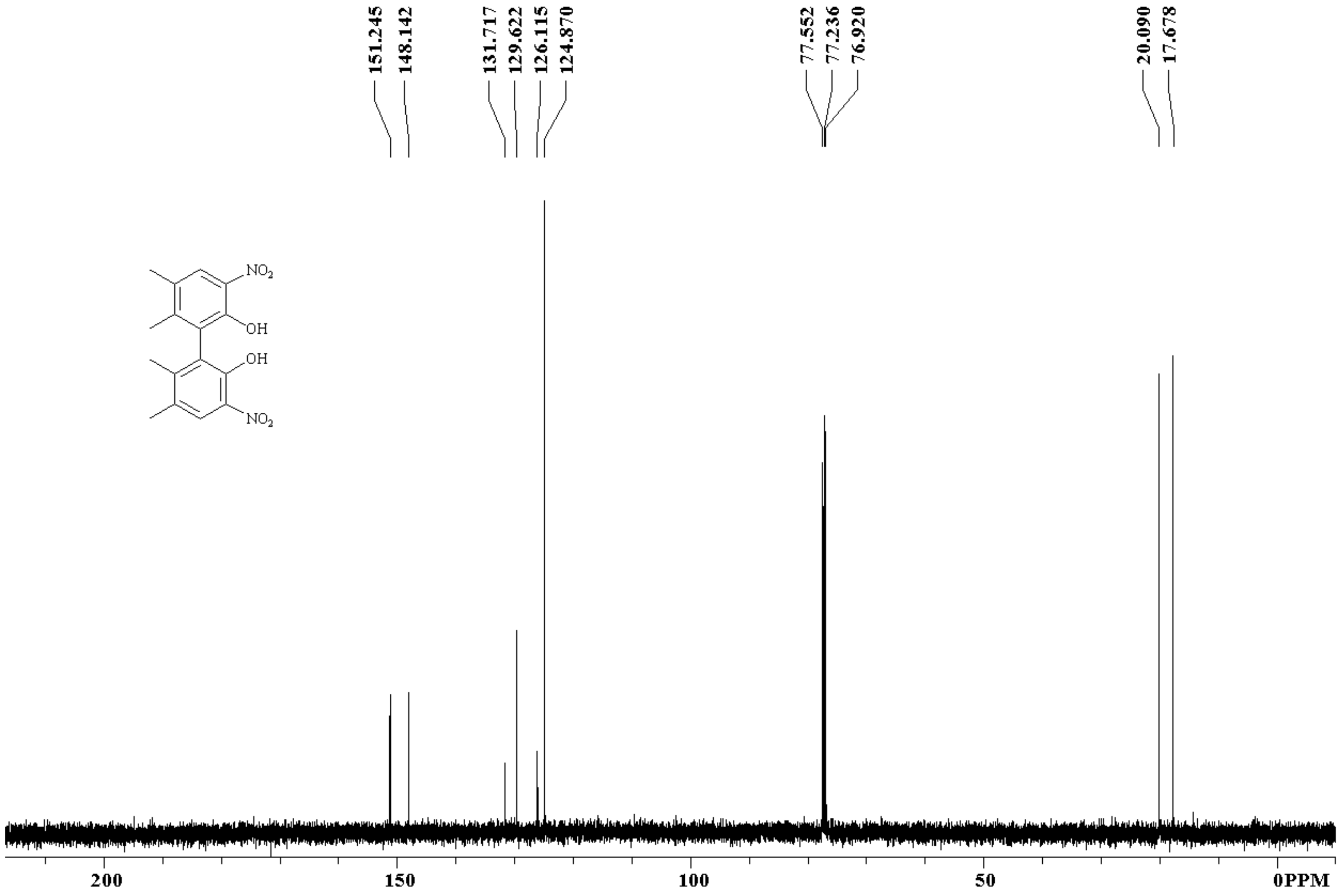
151.245
148.142
131.717
129.622
126.115
124.870
77.552
77.236
76.920
20.090
17.678
NO2
OH
OH
NO2
200
150
100
50
0PPM

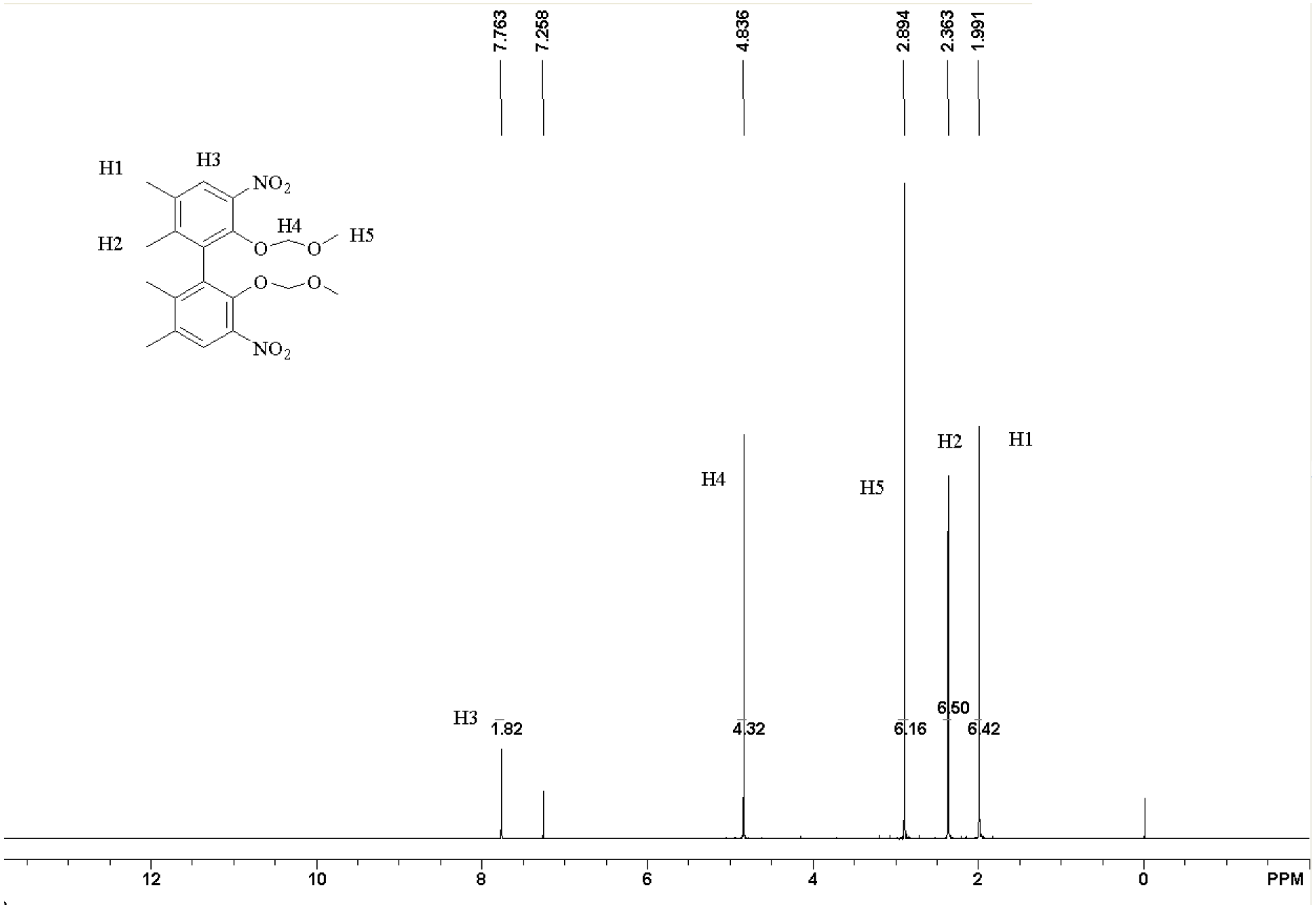
7.763
7.258
4.836
2.894
2.363
1.991
H1
H3
NO_2
H4
H5
H2
O
O
NO_2
H4
H5
H2
H1
H3
1.82
4.32
6.16
6.50
6.42
12
10
8
6
4
2
0
PPM

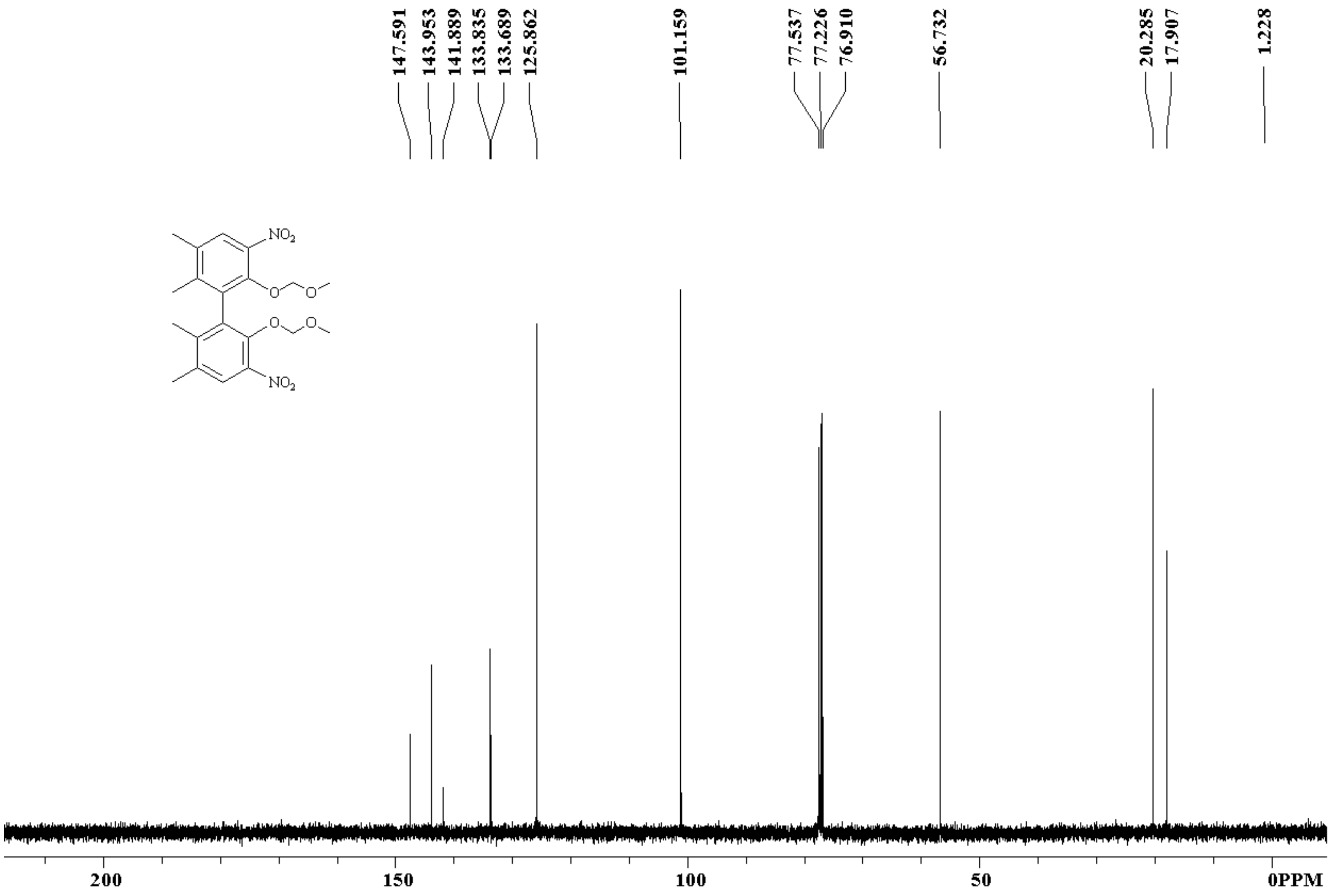
147.591
143.953
141.889
133.835
133.689
125.862
101.159
77.537
77.226
76.910
56.732
20.285
17.907
1.228
200
150
100
50
0PPM
NO2
O
O
O
O
NO2

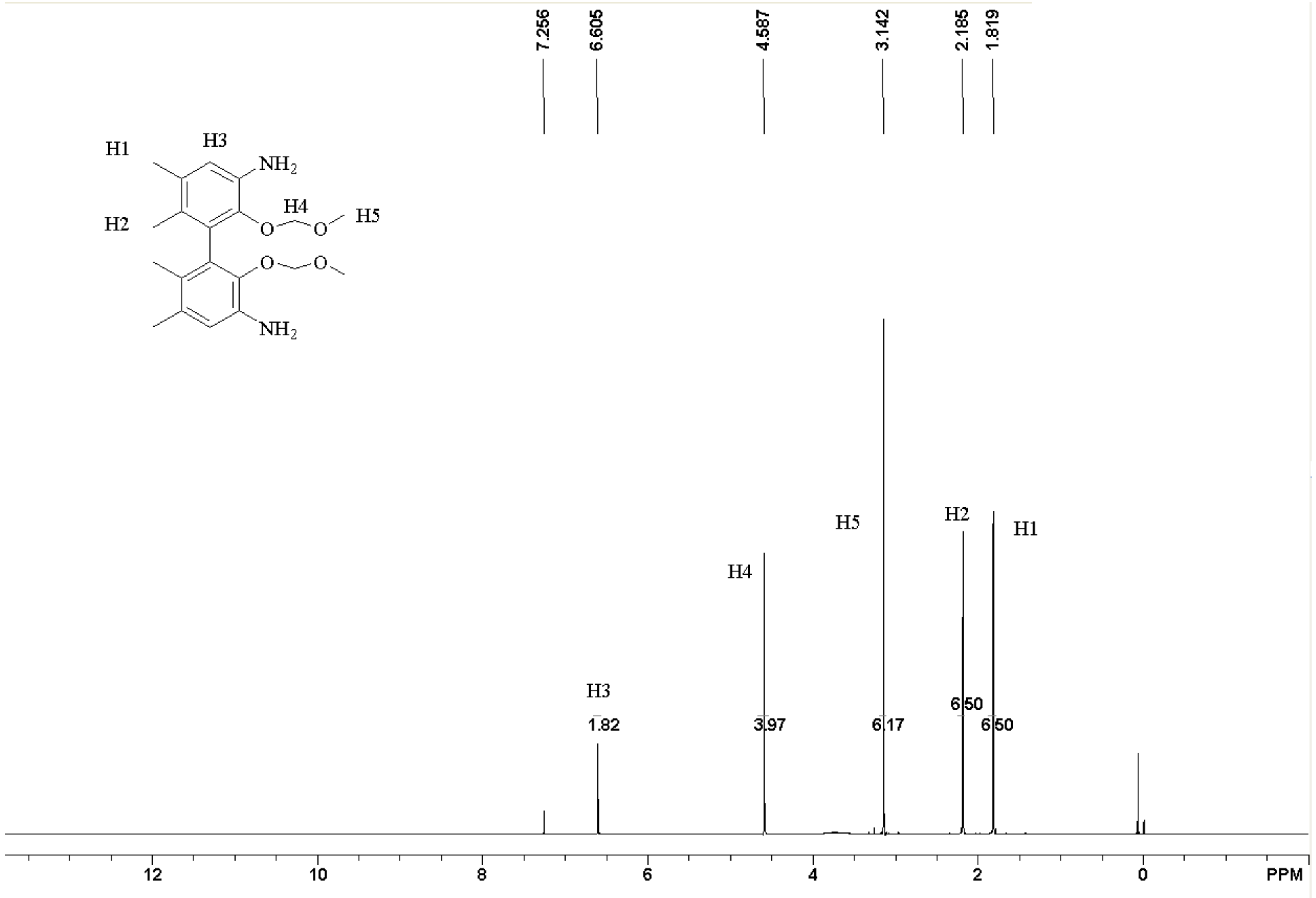

7.256
6.605
4.587
3.142
2.185
1.819
H1
H3
NH_2
H4
H2
H5
NH_2
H5
H2
H1
H4
H3
1.82
3.97
6.17
6.50
6.50
12
10
8
6
4
2
0
PPM

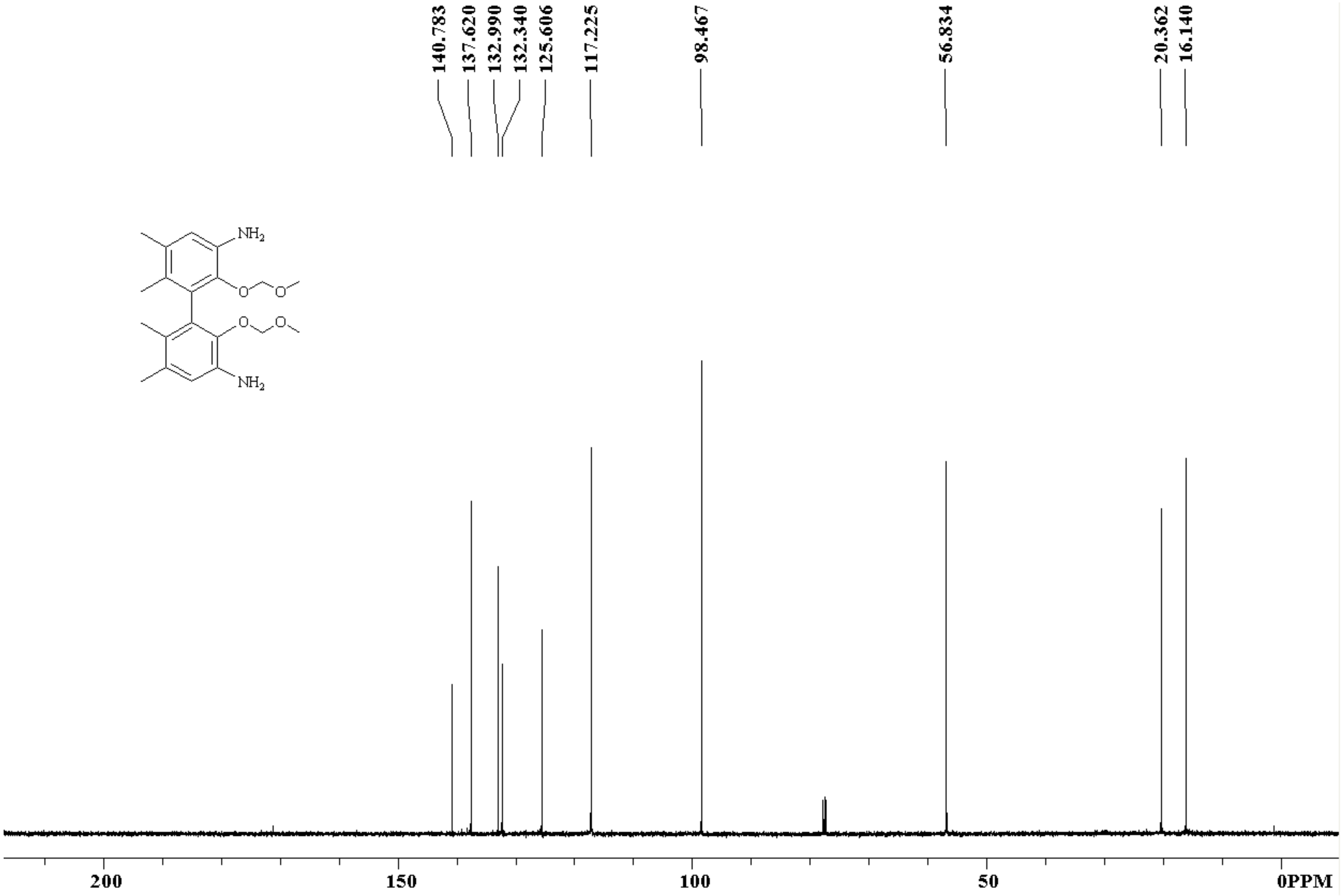
140.783
137.620
132.990
132.340
125.606
117.225
98.467
56.834
20.362
16.140
NH2
O
O
O
O
NH2
200
150
100
50
0PPM

16.2. ***Figures S28*** ^{1}H and ^{13}C NMR of the ligand **L**-(2MOM-2H).

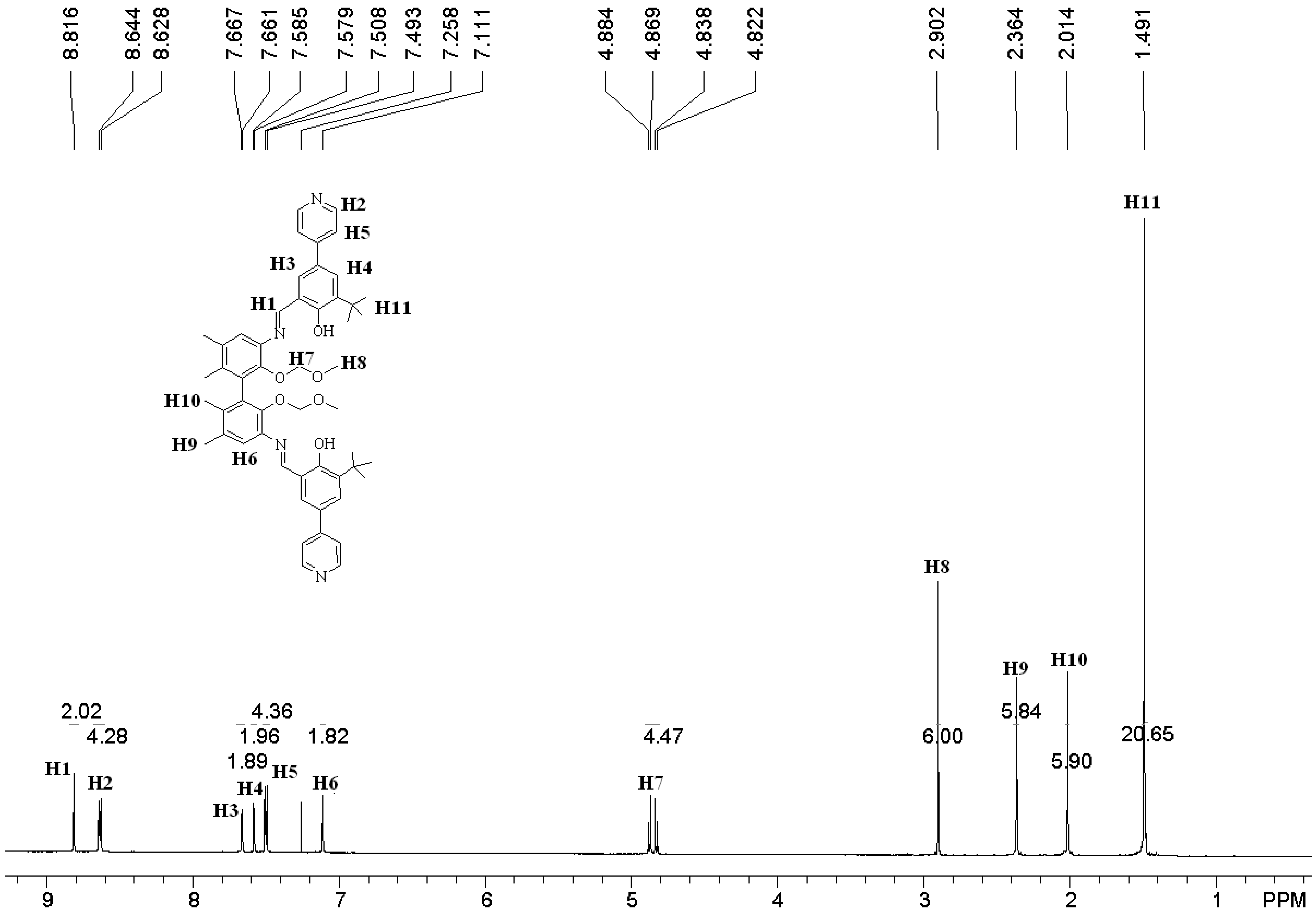

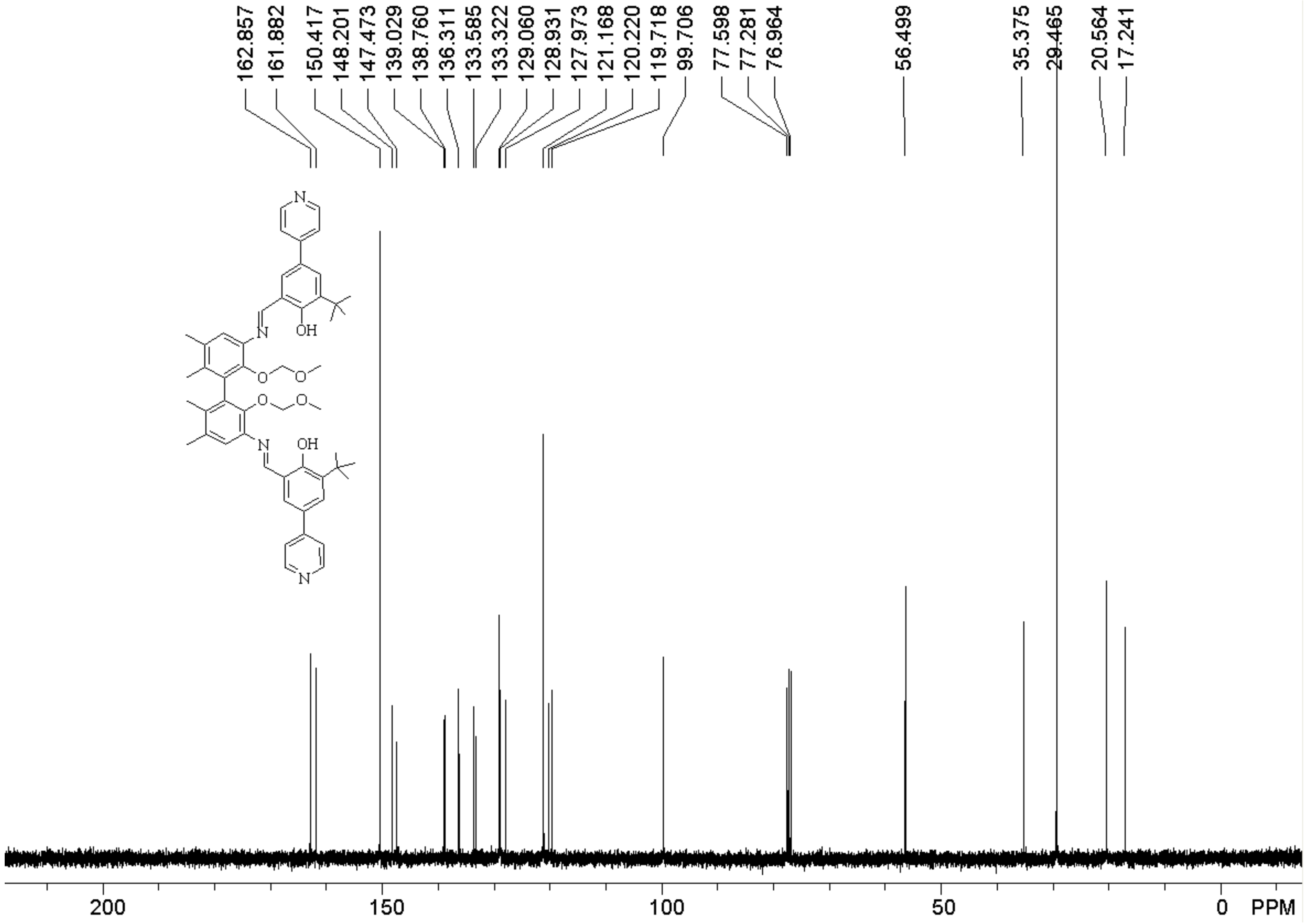
162.857
161.882
150.417
148.201
147.473
139.029
138.760
136.311
133.585
133.322
129.060
128.931
127.973
121.168
120.220
119.718
99.706
77.598
77.281
76.964
56.499
35.375
29.465
20.564
17.241
N
OH
O
N
200
150
100
50
0
PPM

17. ***Figures S29*** UV-Vis Absorption spectra of digested frameworks **2** and **3** loaded with Rhodamine 6G and Brilliant Blue R-250

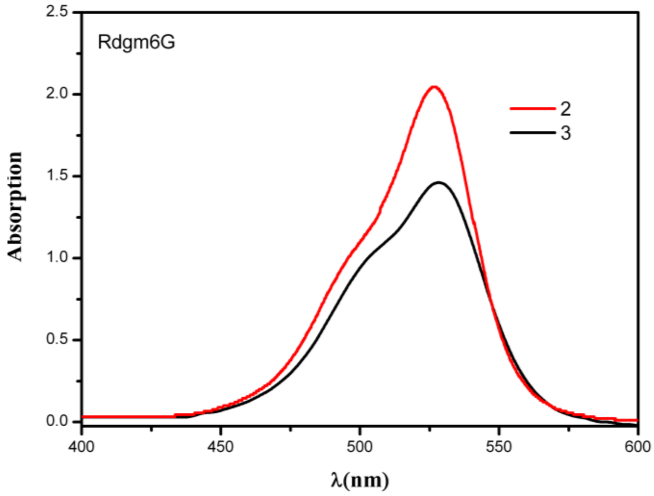

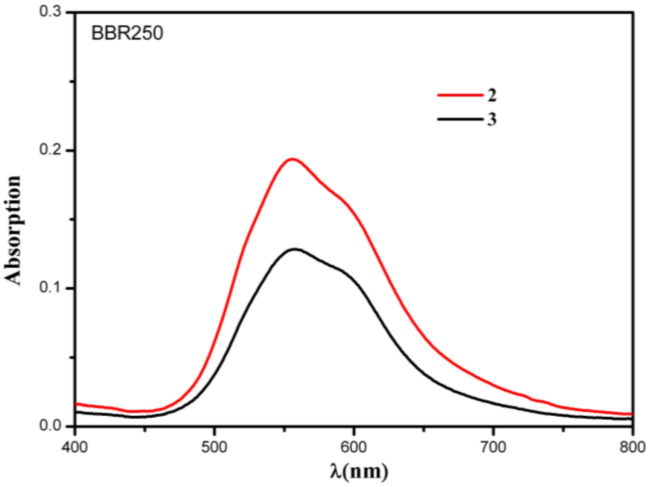

18. ***Figures S30.*** UV-Vis Absorption spectra of Rhodamine 6G and Brilliant Blue R-250. (standard solution)

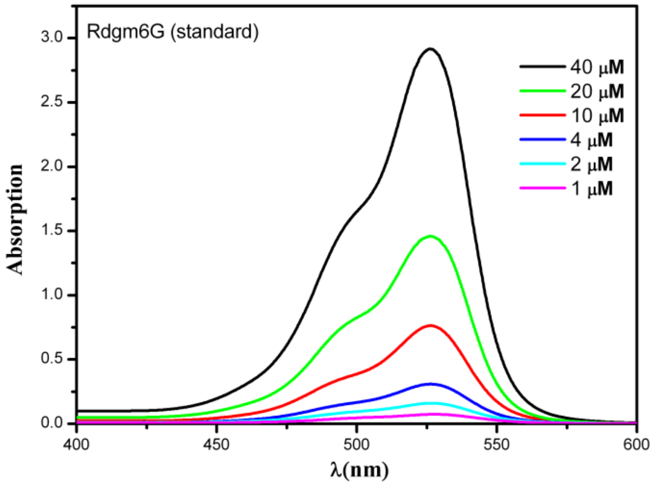

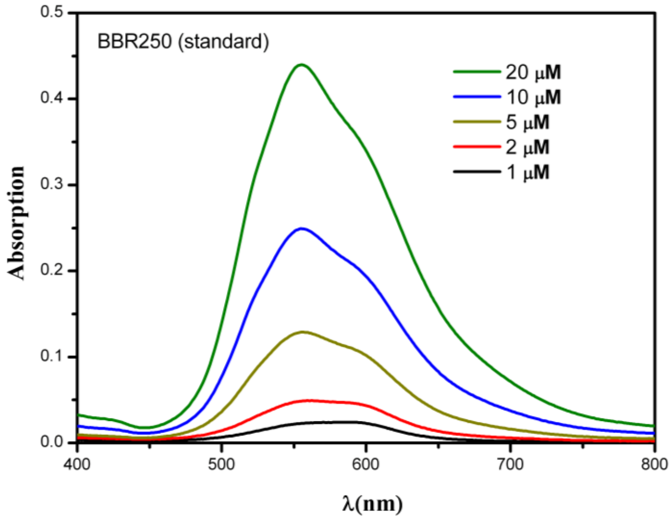

19. ***Figures S31.*** Calibration curve for UV-Vis absorption of Rhodamine 6G and Brilliant Blue R-250

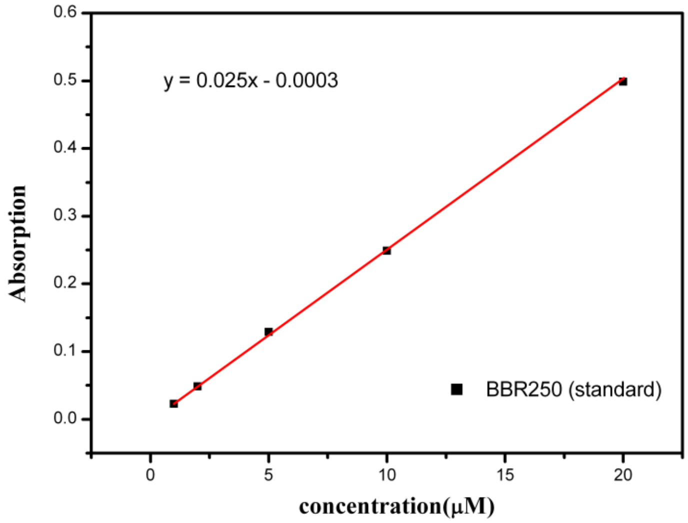

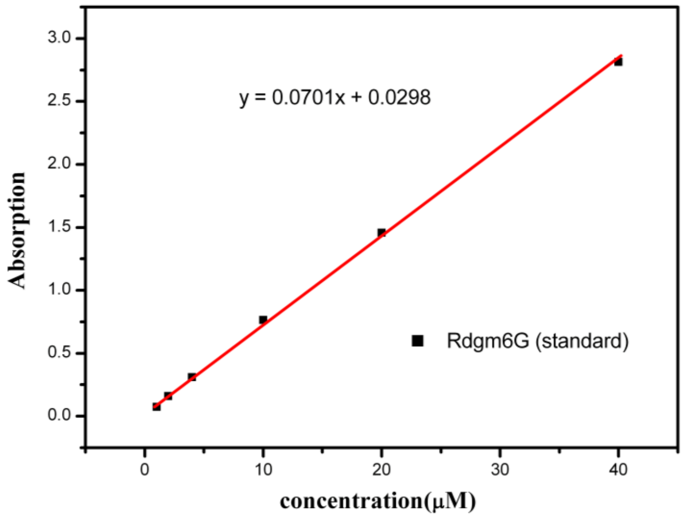

20.*Figures S33*. Powder X-ray diffraction patterns of **2** and **3** with and without Rhodamine 6G and Brilliant Blue R-250

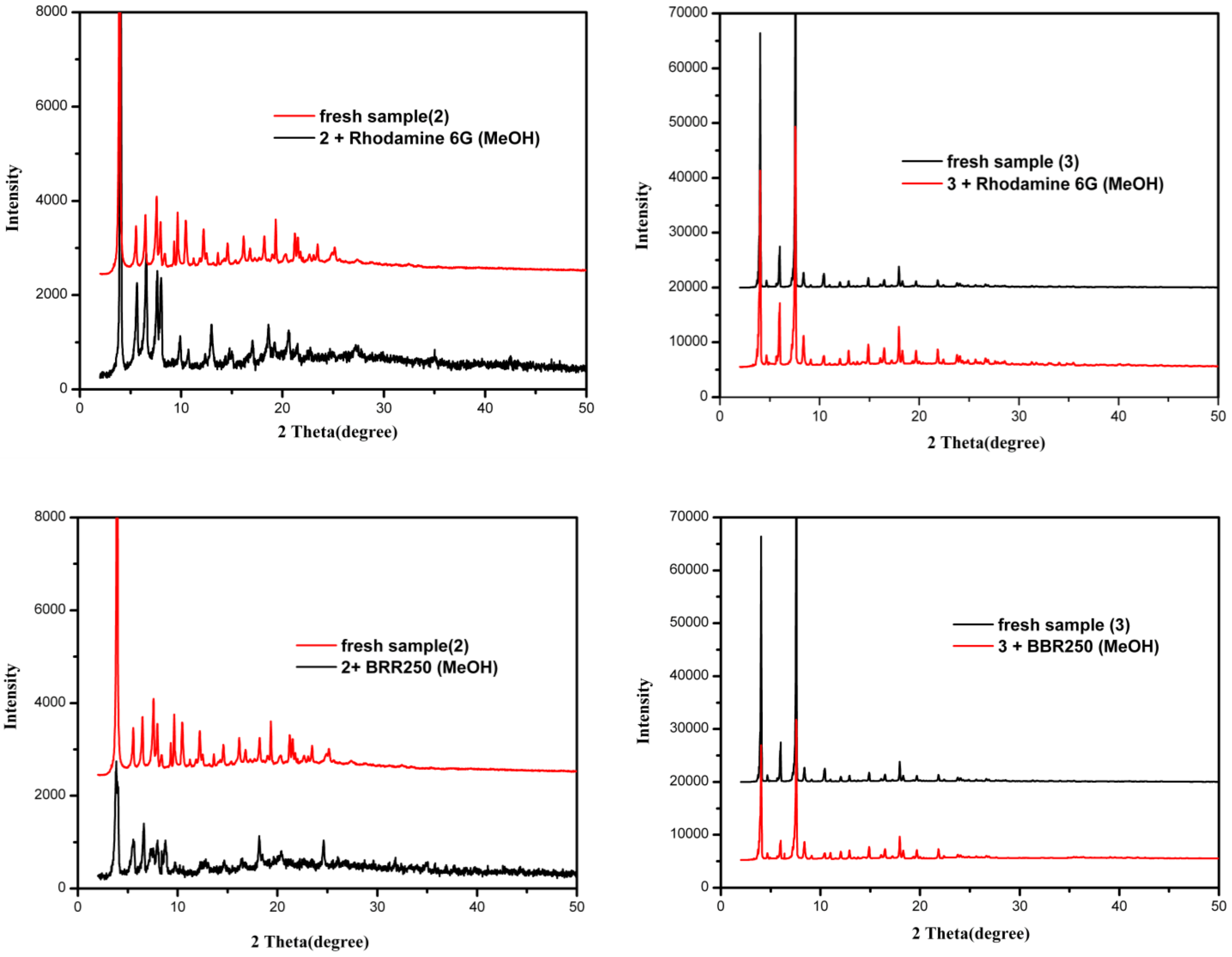

Supplement: Supplementary file 1 [file anie0050-1154-SD1.pdf]
